# The downregulation of SCGN induced by lipotoxicity promotes NLRP3-mediated β-cell pyroptosis

**DOI:** 10.1038/s41420-024-02107-y

**Published:** 2024-07-27

**Authors:** Shuhui Ouyang, Sunmin Xiang, Xin Wang, Xin Yang, Xuan Liu, Meilin Zhang, Yiting Zhou, Yang Xiao, Lingzhi Zhou, Gang Fan, Jing Yang

**Affiliations:** 1 Department of Metabolism and Endocrinology, The First Affiliated Hospital, Hengyang Medical School, University of South China, Hengyang, 421001 Hunan China; 2 Department of Hospital Infection Control, Xingsha District of Hunan Provincial People’s Hospital (Changsha County People’s Hospital), Changsha, 410100 Hunan China; 3 The School of Humanities and Social Sciences, The Chinese University of Hong Kong, Shenzhen, China; 4 Department of pediatrics, Huazhong University of Science and Technology Union Shenzhen Hospital (Shenzhen Nanshan people’s hospital), Shenzhen, 518052 Guangdong China; 5 Department of Urology, Huazhong University of Science and Technology Union Shenzhen Hospital (Shenzhen Nanshan people’s hospital), Shenzhen, 518052 Guangdong China; 6 Department of Metabolism and Endocrinology, Huazhong University of Science and Technology Union Shenzhen Hospital (Shenzhen Nanshan people’s hospital), Shenzhen, 518052 Guangdong China

**Keywords:** Apoptosis, Type 2 diabetes

## Abstract

Lipotoxicity is a well-established phenomenon that could exacerbate damage to islet β-cells and play a significant role in the development of type 2 diabetes, the underlying mechanisms of which, however, remain unclear. In lipotoxic conditions, secretagogin (SCGN), an EF-hand calcium-binding protein abundantly expressed in islets, is found to undergo downregulation. In light of this, we aim to explore the role of SCGN in lipotoxicity-induced β-cell injury. Our findings show that exposure to ox-LDL in vitro or long-term high-fat diets (HFD) in vivo decreases SCGN expression and induces pyroptosis in β-cells. Moreover, restoring SCGN partially reverses the pyroptotic cell death under ox-LDL or HFD treatments. We have observed that the downregulation of SCGN facilitates the translocation of ChREBP from the cytosol to the nucleus, thereby promoting *TXNIP* transcription. The upregulation of TXNIP activates the NLRP3/Caspase-1 pathway, leading to pyroptotic cell death. In summary, our study demonstrates that lipotoxicity leads to the downregulation of SCGN expression in islet β-cells, resulting in ChREBP accumulation in the nucleus and subsequent activation of the NLRP3/Caspase-1 pyroptotic pathway. Thus, administering SCGN could be a potential therapeutic strategy to alleviate β-cell damage induced by lipotoxicity in type 2 diabetes.

## Introduction

Type 2 Diabetes mellitus (T2DM) is a chronic and progressive metabolic disease characterized by insulin resistance, dysfunction of islet β-cells, and ultimately the loss of islet β-cell mass [1]. Hyperlipidemia is a common comorbidity in patients with T2DM [2], and a long-lasting hyperlipidemia may lead to excessive lipid accumulation in pancreatic islets, which could trigger inflammatory injury, impair β-cell function, and even lead to cell death. This phenomenon is defined as lipotoxicity damage [3–5]. Several inflammatory cytokines, including IL-1β, IL-6, Cxcl10, and TNFα, are involved in the development of lipotoxicity-induced β-cell injury, with IL-1β being a crucial role contributor among them [6–9]. Among the signaling pathways involved in the production of IL-1β, the activation of the thioredoxin-interacting protein (TXNIP)/nod-like receptor protein 3 (NLRP3) inflammasome cascade plays a critical role [10]. Recent studies have revealed that glycotoxic injury can activate the NLRP3 inflammasome, leading to β-cell dysfunction and death. Conversely, inhibiting the activation of the NLRP3 inflammasome can protect β-cells and prevent the development of T2DM [11–14]. However, whether lipotoxic injury in β-cells can also activate the NLRP3 inflammasome remains uncertain. Further research is needed to elucidate this potential association.

Secretagogin (SCGN) is an EF-hand calcium (Ca^2+^) binding protein that is highly expressed in pancreatic β-cells and is considered one of the most abundant proteins in these cells, even surpassing the abundance of GAPDH [15]. Previous studies have demonstrated the critical role of SCGN in various aspects of pancreatic β-cell function, including facilitating insulin secretion, regulating α- and β-cell proliferation, and maintaining β-cell specification within the islets [16, 17]. Animal studies have provided compelling evidence that SCGN is expressed in enteroendocrine cells and intestinal neurons. Interestingly, in mice subjected to a high-fat diet (HFD), downregulation of SCGN expression was observed specifically in the islets [18]. This coincided with the presence of inflammation, indicating a potential link between SCGN downregulation and the development of inflammation in the islets [19]. Another recent study investigating an early-onset ulcerative colitis consanguineous family found that functional deficiency of SCGN resulted in increased expression of pro-inflammatory genes in colonic epithelial cells. Similarly, mice lacking SCGN exhibited an upregulation of pro-inflammatory genes in colonic tissues and an enhanced susceptibility to developing colitis when exposed to dextran sulfate sodium salt (DSS) [20]. Given the observed relationship between decreased SCGN expression and heightened inflammatory response in the intestinal tract, we propose that the lipotoxicity-induced downregulation of SCGN may contribute to the promotion of inflammatory injury in β-cells.

Previously, Sorcin, also an EF-hand Ca^2+^-binding protein, has demonstrated its ability to protect against hyperglycemia-induced pancreatic β-cell dysfunction. This protective effect is achieved by retaining the translocation of carbohydrate-responsive element-binding protein (ChREBP) from the cytosol to the nucleus, thereby preserving the transcription of *TXNIP* [21]. As an important islet-enriched transcription factor, ChREBP, has been found to regulate the stress caused by both glucotoxicity and lipotoxicity in islet cells [21–23]. Considering that the intracellular distribution of ChREBP is influenced by Ca^2+^-binding proteins, we hypothesize that SCGN, as the most enriched Ca^2+^-binding protein in pancreatic islets, may also play a role in regulating the distribution and function of ChREBP in pancreatic β-cells. To investigate this hypothesis, we conducted experiments using ox-LDL-treated MIN6 cells and mice models fed with an HFD. Our findings indicate that lipotoxic stimulation induces the downregulation of SCGN and β-cell injury. Furthermore, lipotoxicity-induced downregulation of SCGN promotes the activation of the TXNIP/NLRP3/Caspase-1 pathway, exacerbating NLRP3-mediated β-cell pyroptosis by facilitating the translocation of ChREBP from the cytosol to the nucleus.

## Results

### Ox-LDL treatment triggers pyroptosis in MIN6 cells

**Fig. 1:**
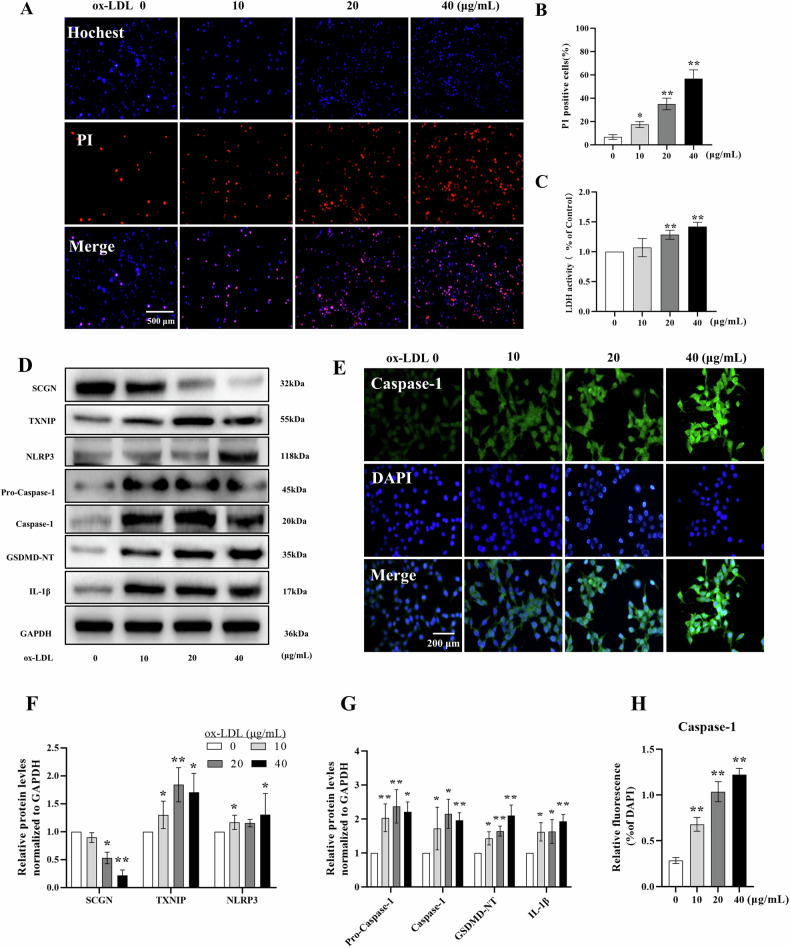
The effects of ox-LDL treatment on MIN6 cell death, SCGN expression, and the activation of the NLRP3/Caspase-1 pathway. **A**–**C** MIN6 cells were treated with increasing concentrations of ox-LDL, and cell death and the release of cellular contents were evaluated using the MTT assay and the LDH release assay. **D**, **F**, **G** Western blot analysis was performed to measure the levels of SCGN, TXNIP, NLRP3, Pro-Caspase-1, Caspase-1, GSDMD-NT, and IL-1β in MIN6 cells treated with increasing concentrations of ox-LDL. **E**, **H** Immunofluorescence staining was used to estimate the protein level of Caspase-1 in MIN6 cells treated with increasing concentrations of ox-LDL. All data were represented as mean ± standard deviation (SD). Scale bars indicate 500 μm (**A**) and 200 μm (**E**). *n* = 3 per group; *P < 0.05, **P < 0.01 compared to the control group.

To investigate the effects of ox-LDL and non-oxidized LDL on SCGN expression in pancreatic β cells, we exposed MIN6 cells to LDL and ox-LDL at a concentration of 40 μg/mL, followed by analyzing the levels of SCGN expression. Our results demonstrated a significant decrease in SCGN expression under both lipotoxic conditions. Notably, ox-LDL induced a slightly more pronounced reduction in SCGN expression compared to non-oxidized LDL (Fig. S1A, B). Based on these findings, we decided to utilize ox-LDL for further in vitro experiments. Subsequently, the MIN6 cells were incubated with increasing concentrations of ox-LDL ranging from 0 to 40 μg/mL. To evaluate cell death and the release of cellular contents, we performed PI staining and an LDH release assay. The results demonstrated that the treatment with ox-LDL promotes concentration-dependent cell death and LDH release in MIN6 cells (Fig. 1A–C). To investigate the potential involvement of SCGN and the proposed NLRP3-mediated inflammatory injury in ox-LDL-induced cell death in MIN6 cells, we examined the protein levels of SCGN and the NLRP3/Caspase-1/GSDMD pyroptotic pathway (Fig. 1D, F, G). Our findings unveiled a significant decrease in SCGN expression, accompanied by a concentration-dependent elevation in the levels of TXNIP, NLRP3, Caspase-1, GSDMD-NT, and IL-1β. To further elucidate the pyroptotic process of MIN6 cells induced by ox-LDL, we performed Caspase-1 fluorescence staining on MIN6 cells treated with ox-LDL. The results depicted in Fig. 1E, H demonstrated a concentration-dependent increase in the fluorescence intensity of Caspase-1 in ox-LDL-treated MIN6 cells. These results suggest that lipotoxic injury can down-regulate SCGN expression and activate pyroptotic cell death in MIN6 cells.

### The islet cells displayed characteristics of pyroptosis in the HFD-treated mice

**Fig. 2:**
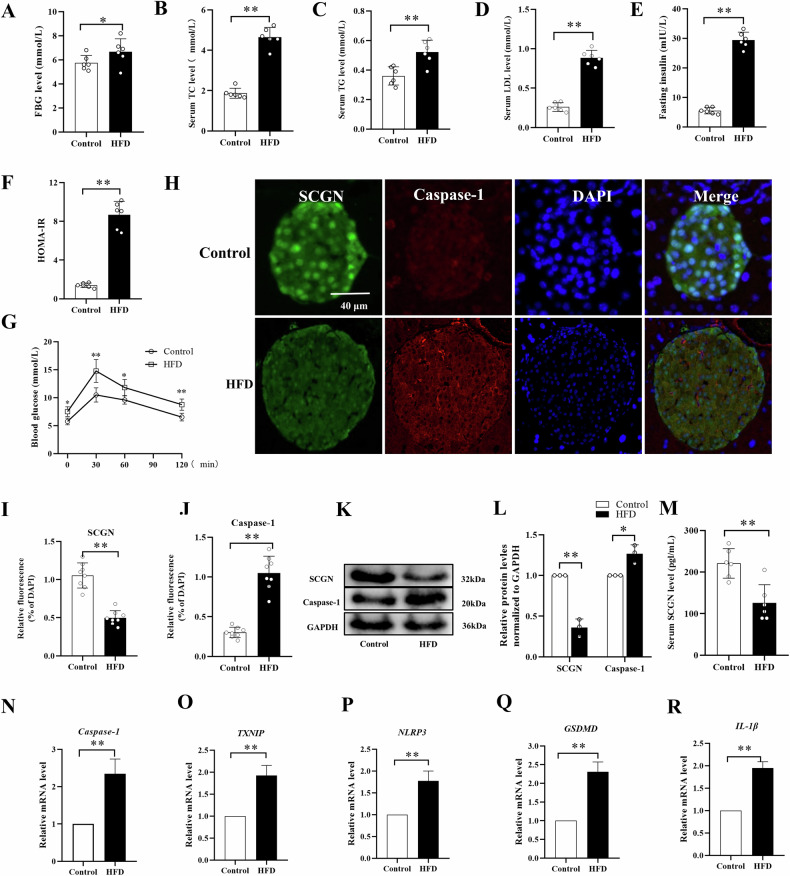
HFD treatment decreases SCGN expression and activates the NLRP3/Caspase-1 pathway in mouse islets. **A**–**D** The serum levels of FBG, TC, TG, and LDL were measured using an automatic biochemical instrument in mice treated with HFD or the normal control diet (*n* = 6 per group). **E**, **F** Fasting insulin levels were measured using an ELISA assay, and the corresponding HOMA-IR values were calculated (*n* = 6 per group). **G** GTT assay was performed in mice after a normal chow diet (control group) or HFD consumption, and the corresponding areas under the curve were calculated. **H**–**L** Immunofluorescence staining and western blot techniques were employed to assess the protein levels of SCGN and Caspase-1 in the islets of both control and HFD-treated mice. **M** The level of SCGN in the serum was measured using an ELISA assay. **N**–**R** Real-time quantitative PCR was used to measure the mRNA levels of Caspase-1, TXNIP, NLRP3, GSDMD-NT, and IL-1β in the islets of HFD-treated mice. All data were presented as mean ± standard deviation (SD). Scale bars = 40 μm (**E**). *n* = 6–8 per group (**A**–**J**, **M**), *n* = 3 per group (**K**, **L**, **N**–**R**); *P < 0.05, **P < 0.01 compared to the control group.

To further investigate the impact of lipotoxicity on pancreatic islets, we conducted an in vivo experiment using mice models treated with an HFD. After a sixteen-week diet intervention, the mice were sacrificed to evaluate the effects. Our findings revealed a significant increase in serum concentrations of fasting blood glucose (FBG), total cholesterol (TC), triglycerides (TG), low-density lipoprotein (LDL), and insulin levels due to the HFD treatment (Fig. 2A–E). Additionally, higher homeostatic model assessment of insulin resistance (HOMA-IR) values was observed (Fig. 2F). Glucose tolerance tests (GTTs) further revealed impaired glucose tolerance in the HFD-treated mice (Fig. 2G). To investigate the impact of an HFD on the expression of SCGN, we assessed the levels of SCGN in both the serum and islets. The findings revealed a noteworthy reduction in SCGN expression in mice subjected to HFD treatment (Fig. 2H, I, K–M). To gain a more comprehensive understanding of the lipotoxic damage inflicted upon islet cells, we conducted Caspase-1 staining within the islets and quantified the mRNA levels of the NLRP3/Caspase-1 pyroptotic pathway. The results revealed a notable elevation in the levels of Caspase-1, TXNIP, NLRP3, GSDMD, and IL-1β within the islets of mice subjected to HFD treatment (Fig. 2H, J, N–R). This indicates a significant upregulation of these pyroptotic pathway markers, which are closely linked to lipotoxic β-cell injury.

### Knockdown of *SCGN* replicated the effects of ox-LDL treatment on pyroptotic cell death

To investigate the role of *SCGN* in cell behavior, we employed a specific *SCGN* siRNA technique to silence SCGN expression in MIN6 cells. Our aim was to examine the impact of *SCGN* depletion on cell viability and the activation of the NLRP3/Caspase-1 pyroptotic pathway. In a previous study [24], we evaluated three siRNA candidates and identified the most efficient siRNA with the highest silencing efficiency. In this current study, we reaffirmed its effectiveness in suppressing *SCGN* expression (Fig. S1A–C).

**Fig. 3:**
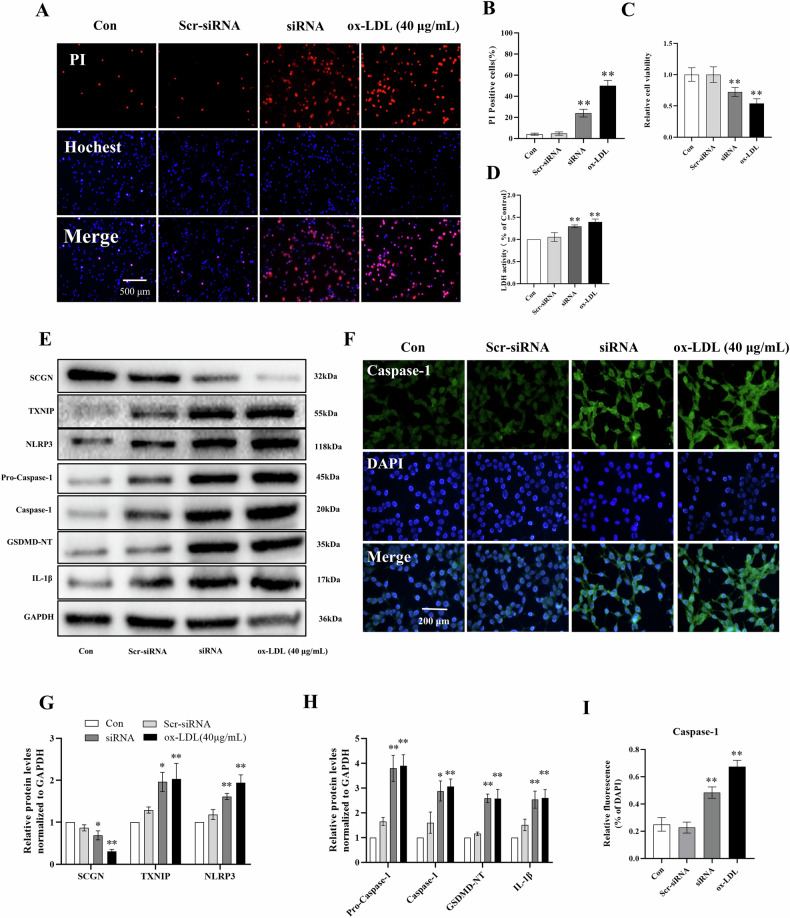
The knockdown of SCGN in MIN6 cells replicated the effects of ox-LDL treatment on pyroptotic cell death. **A**–**D** The impact of SCGN siRNA or ox-LDL on cell viability, cell death, and the release of cellular contents was evaluated using PI staining, MTT assay, and LDL release assay, respectively. **E**, **G**, **H** The protein levels of SCGN, TXNIP, NLRP3, Pro-Caspase-1, Caspase-1, GSDMD-NT, and IL-1β in MIN6 cells were measured through western blot analysis post-SCGN siRNA or ox-LDL treatment. **F**, **I** The level of Caspase-1 in MIN6 cells following SCGN siRNA or ox-LDL treatment was also estimated via Immunofluorescence staining. All data were presented as mean ± standard deviation (SD). The scale bars represent 500 μm (**A**) and 200 μm (**F**). Statistically significant differences were denoted by *P < 0.05, **P < 0.01 compared to the control group, *n* = 3 per group.

Conceivably, the silencing of *SCGN* using siRNA in MIN6 cells resulted in significant inhibition of cell proliferation, leading to subsequent cell death and the release of LDH, which resembled the effects observed when the cells were treated with ox-LDL (Fig. 3A–D). The findings suggest that SCGN plays a crucial role in promoting cell survival and proliferation in MIN6 cells, and SCGN may play a potential role in mediating cellular responses associated with ox-LDL-induced cytotoxicity.

Additionally, we performed Western blot and immunofluorescence analyses, revealing that *SCGN* silencing triggered the activation of the NLRP3/Caspase-1 pyroptotic pathway. Notably, these effects closely mirrored the cellular responses induced by ox-LDL treatment (Fig. 3E–I). This discovery strengthens the link between SCGN and the pyroptotic pathway, emphasizing its potential involvement in the cellular mechanisms triggered by exposure to ox-LDL.

### SCGN overexpression partly rescued ox-LDL-induced pyroptosis

**Fig. 4:**
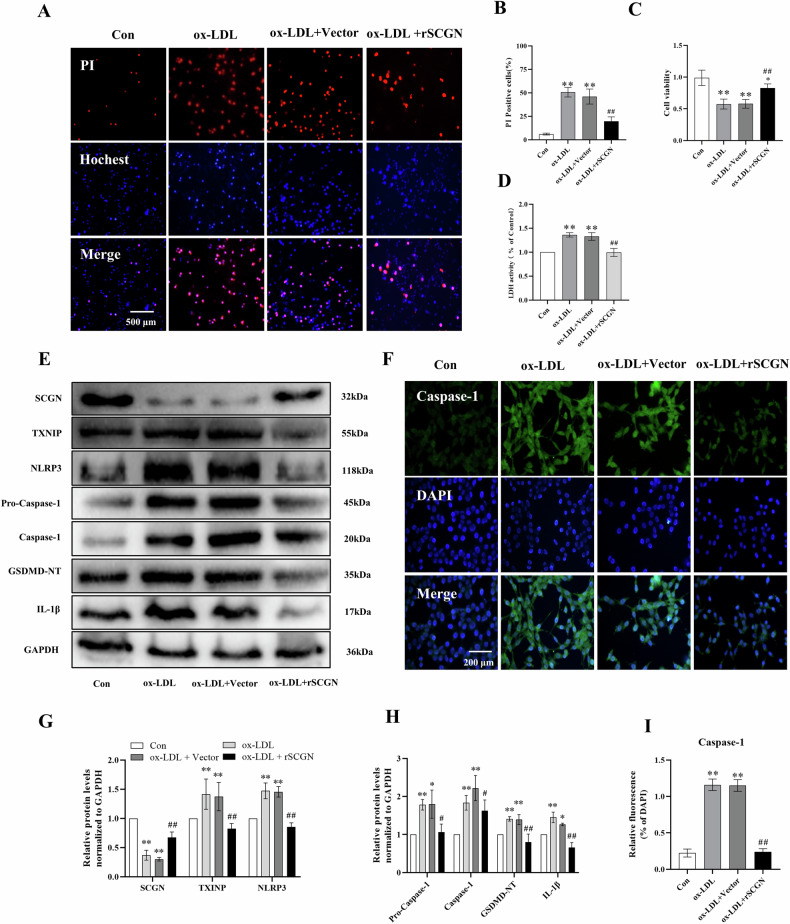
Overexpression of SCGN partially rescued ox-LDL-induced pyroptosis in MIN6 cells. **A**–**D** PI staining, MTT assay, and LDL release assay were used to assess cell death, cell viability, and release of cellular contents in MIN6 cells treated with ox-LDL or rSCGN in combination with ox-LDL. **E**, **G**, **H** Western blot analysis was performed to measure the protein levels of SCGN, TXNIP, NLRP3, Pro-Caspase-1, Caspase-1, GSDMD-NT, and IL-1β in MIN6 cells treated with ox-LDL or rSCGN in combination with ox-LDL. **F**, **I** Immunofluorescence staining was used to estimate the level of Caspase-1 in MIN6 cells following treatment with ox-LDL or rSCGN in combination with ox-LDL. All data were presented as mean ± standard deviation (SD). Scale bars represent 500 μm (**A**) and 200 μm (**F**). *P < 0.05, **P < 0.01 compared to the control group; ^#^*P* < 0.05, ^##^*P* < 0.01 compared to the ox-LDL or ox-LDL in combination with control vector treated groups, *n* = 3 per group.

To further confirm the protective effect of SCGN in ox-LDL-treated MIN6 cells, we conducted functional recovery experiments using a mouse-origin recombinant pcDNA3.1-SCGN vector (rSCGN). The results demonstrated that pretreatment of MIN6 cells with rSCGN partially rescued MIN6 cells from ox-LDL-induced cell death and significantly reduced the release of LDH (Fig. 4A–D). Furthermore, our mechanistic investigations revealed that SCGN had a profound impact on the cellular pathway involved in pyroptosis (Fig. 4E–I). Specifically, it inhibited the activation of the NLRP3/Caspase-1 pyroptotic pathway. This finding provides crucial insights into the underlying molecular mechanisms by which SCGN exerts its protective effects against ox-LDL-induced cell damage in MIN6 cells, specifically by mitigating cell pyroptosis.

### SCGN alleviates HFD-induced islet pyroptosis in mice

**Fig. 5:**
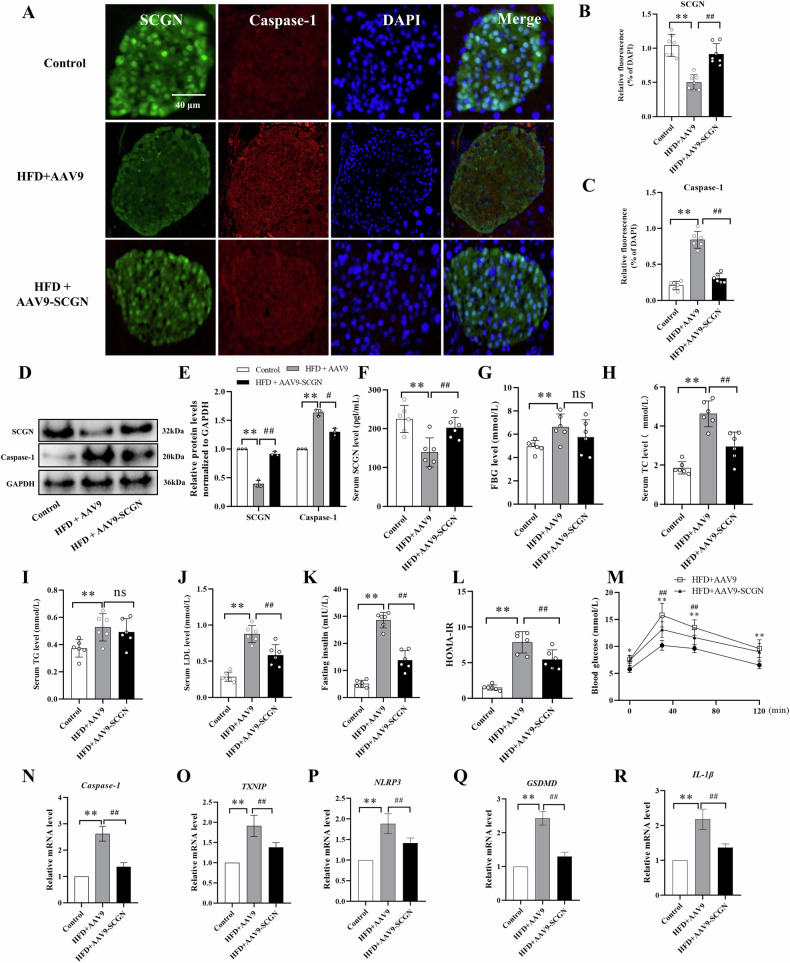
SCGN treatment improves HFD-induced hyperlipidemia and protects against islet pyroptosis. **A**–**E** Immunofluorescence staining and western blot analysis were employed to assess the protein levels of SCGN and Caspase-1 in the islets of mice subjected to three different conditions: normal chow diet, HFD in combination with AAV-9 (HFD + AAV-9), or HFD in combination with AAV-9-*SCGN* (HFD + AAV-9-*SCGN*). **F** The level of SCGN in the serum was measured using an ELISA assay. **G**–**J** The serum levels of FBG, TC, TG, and LDL were quantified using an automatic biochemical instrument in mice exposed to the three conditions. **K**, **L** Fasting insulin levels were determined using an ELISA assay, and the corresponding HOMA-IR values were calculated based on these results and the FBG levels. **M** GTT assay was performed in each group of mice, and the corresponding areas under the curve were calculated. **N**–**R** Real-time quantitative PCR was employed to assess the mRNA levels of Caspase-1, TXNIP, NLRP3, GSDMD-NT, and IL-1β in the islets of mice subjected to the three conditions. All data were presented as mean ± standard deviation (SD). Scale bars = 40 μm (**A**). *n* = 6–7 per group (**A**–**C**, **F**–**M**), *n* = 3 per group (**D**, **E**, **N**–**R**); *P < 0.05, **P < 0.01 compared to the control group, ^#^*P* < 0.05, ^##^*P* < 0.01 compared to the HFD + AAV-9 group.

To further observe the protective effect of SCGN against lipotoxic islet injury, we utilized recombinant AAV-9 to deliver *SCGN* in a mouse model that was treated with an HFD. To confirm the efficacy of AAV-9-*SCGN* treatment in increasing SCGN levels, we performed a series of experiments, including immunofluorescence staining, western blot analysis, and serum SCGN ELISA assays. Our results unequivocally demonstrated the successful upregulation of SCGN in mice treated with AAV-9-*SCGN*. (Fig. 5A–F). Subsequently, we conducted blood biochemistry tests to evaluate the effect of AAV-9-*SCGN* treatment on various metabolic parameters. Our findings exhibited a significant improvement in the elevated levels of FBG, TC, TG, LDL, and fasting insulin induced by the HFD treatment (Fig. 5G–K). Consequently, this led to a decrease in the HOMA-IR values (Fig. 5L). GTTs further revealed that AAV-9-*SCGN* treatment improved impaired glucose tolerance compared to AAV-9 control mice after HFD feeding (Fig. 5M). Furthermore, relative semi-quantitative immunofluorescence staining and RT-qPCR results provided valuable insights into the underlying mechanisms of SCGN’s protective effects. They revealed that SCGN restoration reduced the activation of the NLRP3/Caspase-1 pyroptotic pathway, this was supported by a notable decrease in the levels of Caspase-1, TXNIP, NLRP3, GSDMD, and IL-1β within the islets of mice treated with an HFD (Fig. 5A, C, N–R). This finding highlights SCGN as a potential therapeutic target in mitigating lipotoxicity-induced inflammation and pyroptotic cellular damage in islet cells.

### SCGN inhibits the translocation of ChREBP from the cytosol to the nucleus

**Fig. 6:**
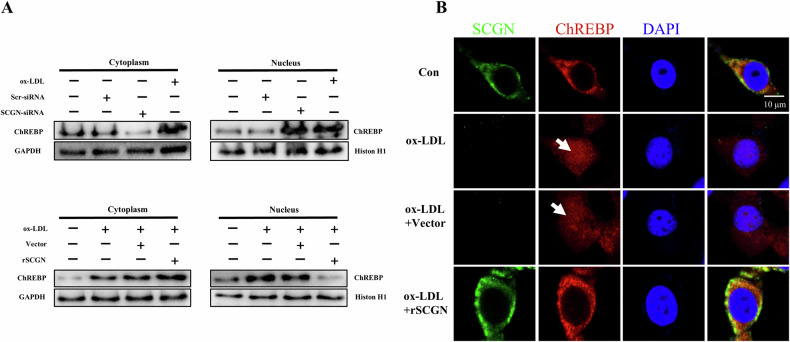
SCGN inhibits the translocation of ChREBP from the cytosol to the nucleus in β-cells. **A** The levels of ChREBP in the cytoplasm and nucleus were assessed by western blot analysis in β-cells treated with ox-LDL, SCGN siRNA, or rSCGN in combination with ox-LDL. **B** Immunofluorescence staining was used to measure the levels of SCGN (green) and ChREBP (red) in β-cells treated with ox-LDL, SCGN siRNA, or rSCGN in combination with ox-LDL. *n* = 3 per group; Scale bars = 10 μm.

Previous studies have shown that glucotoxicity or lipotoxicity stress can lead to the translocation of ChREBP from the cytosol to the nucleus, promoting the transcription of *TXNIP* in cells. As the intracellular distribution of ChREBP is influenced by Ca^2+^-binding proteins, we hypothesized that SCGN may regulate the distribution of ChREBP in β-cells in response to lipotoxicity. To test this hypothesis, we examined the subcellular localization of ChREBP in MIN6 cells using western blot analysis. We observed that both ox-LDL and *SCGN* siRNA treatment led to an increased accumulation of ChREBP in the nucleus, while rSCGN treatment effectively preserved the cytosolic abundance of ChREBP (Fig. 6A). This finding was further confirmed by double staining of SCGN and ChREBP (Fig. 6B) in MIN6 cells treated with ox-LDL or ox-LDL in combination with rSCGN.

**Fig. 7:**
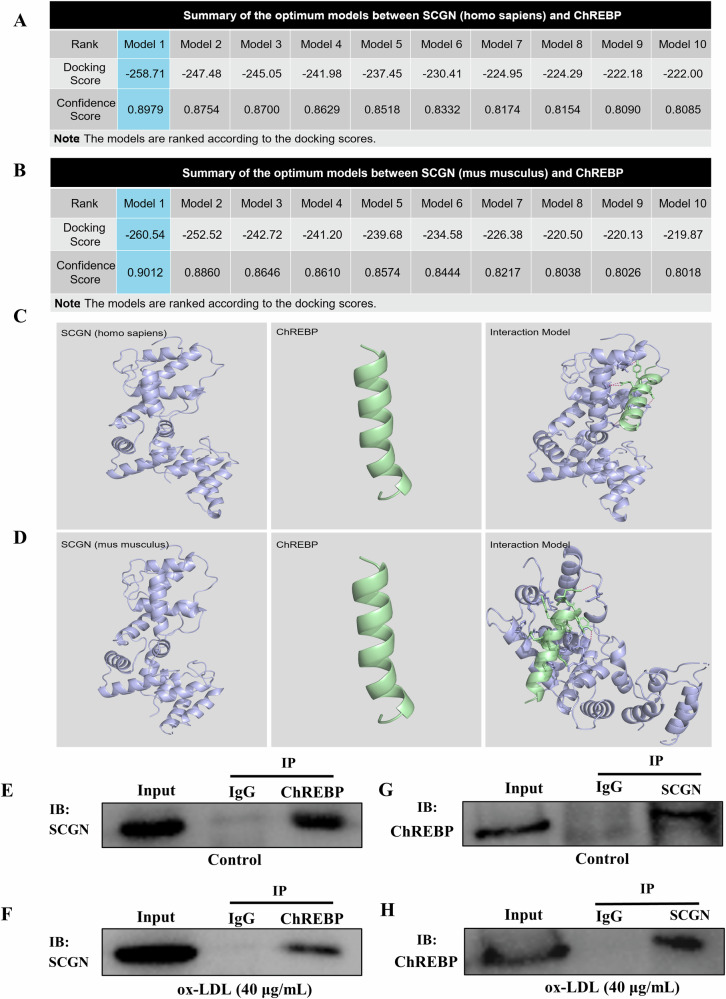
SCGN demonstrates a direct binding affinity for the ChREBP protein. **A**, **B** Bioinformatics analysis results indicate that the docking score in the top ten predicted models between SCGN and ChREBP are all below −200, with the lowest value −258.71 in homo sapiens and −260.54 in mouse models. Likewise, the confidence scores are above 0.7, with a pronounced value of 0.89 in homo sapiens and 0.90 in mouse models. **C**, **D** The binding capability of the two proteins were further analyzed and visualized by the Pymol software. **E**–**H** Co-IP experiment confirmed the binding relationship between SCGN and ChREBP under both normal and ox-LDL-treatment conditions.

Given that a reduction in SCGN levels enhances the nuclear accumulation of ChREBP, we proceeded to investigate the potential interaction between SCGN and ChREBP. In a protein docking analysis conducted using the HDOCK server, we found that the docking scores between SCGN and ChREBP in the top ten predicted models are all below −200, with the lowest value of −258.71 in human and −260.54 in mouse models. Likewise, the confidence scores are above 0.7, with the highest value of 0.89 in humans and 0.90 in mouse models (Fig. 7A, B). Then, the binding capability of the two proteins were further analyzed and visualized by the Pymol software (Fig. 7C, D). These in silico analysis of protein–protein interactions predicted that SCGN has the ability to bind with ChREBP protein. Subsequently, a co-immunoprecipitation assay experiment was conducted to further confirmed the interaction between the two proteins, As expected, SCGN was found to bind with ChREBP under both normal and ox-LDL-treatment conditions (Fig. 7E–H). Overall, our study provides evidence supporting the idea that SCGN is involved in regulating the intracellular distribution of ChREBP in β-cells when exposed to lipotoxic conditions. This regulatory role likely involves the prevention of ChREBP translocation to the nucleus.

## Discussion

Although the detrimental effects of lipotoxicity on islet β cells are well-known, the specific mechanisms involved in this process have not been extensively investigated. In this study, we focused on examining the potential regulatory role of SCGN, a calcium-binding protein abundantly present in islets, in mitigating the cellular stress induced by lipotoxicity. By conducting a series of in vitro and in vivo experiments, we successfully elucidated the crucial involvement of SCGN in modulating this process. In MIN6 cells, the expression of SCGN was significantly reduced when treated with ox-LDL. Moreover, the treatment with ox-LDL led to a concentration-dependent rise in cell death and LDH release. Additionally, there was a notable decline in SCGN expression along with the activation of NLRP3/Caspase-1 pyroptotic pathway proteins as a consequence of the ox-LDL treatment. Interestingly, when the expression of SCGN was silenced, it replicated the effects of ox-LDL treatment by inducing pyroptotic cell death. On the contrary, treatment with rSCGN partially attenuated ox-LDL-induced cell pyroptosis by suppressing the activation of the NLRP3/Caspase-1 pyroptotic pathway. These findings suggest that treatment with ox-LDL leads to a reduction in SCGN levels in MIN6 cells, consequently activating the NLRP3/Caspase-1 pyroptotic pathway, which, ultimately, triggers inflammation and cell death.

In line with our in vitro experiments and previous studies [18, 25], we further revealed a remarkable reduction in SCGN expression both in the serum and islets of mice exposed to an HFD treatment. Additionally, our in vivo investigations also demonstrated a significant upregulation of NLRP3/Caspase-1 pyroptotic pathway proteins in the islets of HFD-treated mice. However, a remarkable reversal occurred when we employed AAV-9-mediated *SCGN* restoration, as it effectively rescued the HFD-induced activation of the NLRP3/Caspase-1 pyroptotic pathway. In alignment with another study [26], our research further revealed that SCGN restoration had a positive impact on mitigating the rise in both hyperglycemia and hyperlipidemia. Consequently, this led to the improvement of insulin resistance in mice that were subjected to an HFD. These findings indicate that SCGN plays a critical role in protecting islets from injury and in maintaining metabolic homeostasis.

Activation of the NLRP3/Caspase-1 pyroptotic pathway requires the presence of TXNIP. TXNIP plays a crucial role in regulating the inflammasome complex formation and activation, specifically the NLRP3 inflammasome [27, 28]. When cellular stress occurs, such as during high glucose treatment, TXNIP binds to NLRP3 and promotes the assembly of the NLRP3 inflammasome. This, ultimately, leads to the activation of Caspase-1, initiating the process of pyroptosis [28, 29]. The expression level of TXNIP in islet cells is under the regulation of ChREBP, a transcription factor known for its significant role in glucose metabolism and lipogenesis [30, 31]. When glucose levels are elevated, ChREBP is activated and translocated to the nucleus, where it binds to the carbohydrate response element (ChoRE) present in the promoter region of the *TXNIP* gene [21]. This binding activates the transcription of the *TXNIP* gene, leading to an increase in TXNIP expression in islet cells. Elevated glucose levels have been demonstrated to stimulate the nuclear accumulation of ChREBP by inducing Ca^2+^ influx. Consequently, this leads to conformational changes in sorcin protein and its subsequent translocation from the cytosol to the membranes, where it interacts with target proteins. This process can be prevented by chelating extracellular Ca^2+^ or using pharmacological inhibitors to block Ca^2+^ entry [21, 22, 32]. Given that SCGN is also a Ca^2+^-binding protein, it undergoes conformational changes that are dependent on the presence of Ca^2+^. These changes enable SCGN to activate its function in response to intracellular stress [33]. Based on this information, we predicted that SCGN could potentially play a crucial role in modulating the translocation of ChREBP from the cytosol to the nucleus. This suggested mechanism may provide a protective effect against lipotoxicity. Indeed, in our study, we observed a interaction between SCGN and ChREBP, and the ox-LDL-induced SCGN decrease could promote the translocation of ChREBP from the cytosol to the nucleus.. We have reached a preliminary conclusion indicating that SCGN plays a significant role in regulating the intracellular distribution of ChREBP in β-cells in response to lipotoxicity, likely by impeding its translocation to the nucleus.

In addition to the protective effect of SCGN against pancreatic lipotoxicity, our in vivo experiments also revealed its potential to ameliorate dyslipidemia and insulin resistance in mice subjected to an HFD. Although the specific mechanisms underlying the improvement in blood lipid levels and insulin resistance were not explored in this study. Previous research has indicated that circulating SCGN can enter cells lacking SCGN expression, such as smooth muscle cells and liver cells, via endocytosis [34]. Furthermore, administration of exogenous SCGN has been found to enhance insulin signaling pathway activity, consequently alleviating insulin resistance [16]. These findings suggest a potential role for SCGN as a therapeutic agent for metabolic disorders characterized by dyslipidemia and insulin resistance. In this study, we observed a significant increase in SCGN expression in islets after infecting mice with AAV-9-*SCGN* virus, as well as a significant elevation in the level of circulating SCGN. Therefore, it is plausible that the increased circulating SCGN enters other tissues beyond the islets via endocytosis, contributing to the improvement of insulin resistance. Further investigations are needed to elucidate the precise mechanisms involved.

Nonetheless, it is important to acknowledge the limitations of our study. Currently, the understanding of the interaction between SCGN and ChREBP is primarily focused on changes in ChREBP distribution between the cytosol and the nucleus in response to fluctuations in intracellular SCGN levels, as well as through in silico prediction and co-IP assays. To fully elucidate this intricate interaction, additional investigations are needed. This could include investigating the specific binding domains involved and exploring the impact of changes in cytosolic Ca^2+^ concentration on this process. Additionally, more investigations are needed to elucidate how ox-LDL accumulation decreases SCGN expression and whether the SCGN-regulated NLRP3/Caspase-1 pathway is impaired in patients with hypercholesterolemia. Moreover, it would be advantageous to examine the changes in SCGN expression in tissues other than islets following AAV-9-mediated SCGN overexpression. Such investigations would provide a deeper understanding of the mechanisms through which SCGN improves insulin resistance and lipid disorders.

## Conclusion

**Fig. 8:**
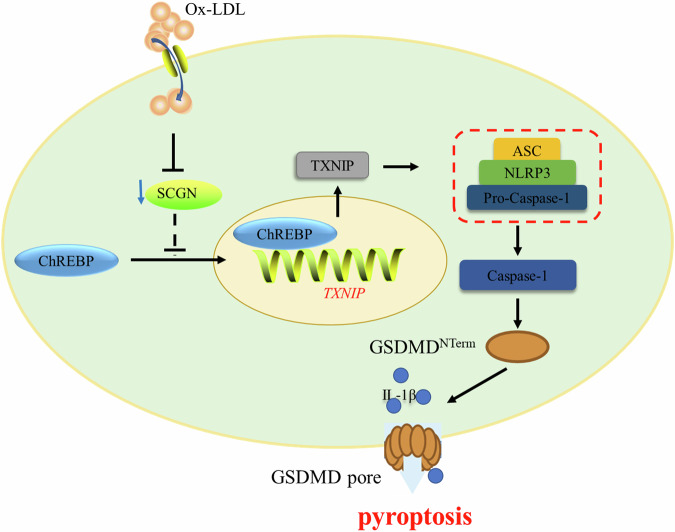
Schematic diagram illustrating the SCGN-regulated mechanism of pyroptosis in MIN6 cells treated with ox-LDL. Ox-LDL treatment causes a decrease in SCGN expression in MIN6 cells. The downregulation of SCGN facilitates the translocation of ChREBP from the cytosol to the nucleus, resulting in increased transcription of TXNIP. Increased expression of TXNIP activates the NLRP3/caspase-1 pyroptotic pathway, leading to β cell pyroptosis.

This study provides novel evidence for the harmful impact of lipotoxic injury on pancreatic β-cells, resulting in the downregulation of SCGN expression. This reduction in SCGN expression facilitates the translocation of ChREBP from the cytosol to the nucleus, promoting *TXNIP* transcription and triggering the activation of the NLRP3/caspase-1 pyroptotic pathway, ultimately leading to pyroptotic pancreatic β-cell death (Fig. 8). Our findings shed light on the intricate interplay between lipotoxicity, SCGN expression, and the NLRP3 inflammasome, thereby enhancing our understanding of the pathogenesis of lipotoxicity-induced pancreatic β-cell injury. Such insights may pave the way for the development of new therapeutic strategies to combat pancreatic disorders related to lipotoxicity and inflammatory processes.

## Methods

### Antibodies and reagents

The following primary antibodies were used in this study: TXNIP (18243-1-AP) and NLRP3 (19771-1-AP) from Proteintech (Wuhan, Hubei, China), ChREBP (NB400-135) from Novusbio (Centennial, CO, USA), GSDMD (ab138483) from Santa Cruz Biotechnology (Santa Cruz, CA, USA), Caspase-1 (ab207802), GAPDH (ab245355), IL-1β (ab234437), Histone1 (ab8898), and SCGN (ab137017) from Abcam (Cambridge, UK). The goat anti-rabbit IgG secondary antibody (ab205718) was obtained from Abcam. SCGN-specific siRNAs and scrambled siRNAs were procured from RiboBio (Guangzhou, China). A pcDNA3.1 vector containing the complete coding sequence region of mouse Scgn (NM_145399) was constructed by Gene Chem (Shanghai, China). Additionally, Gene Chem also constructed adeno-associated virus serotype 9 (AAV-9) vectors containing the full-length coding sequence of Scgn. A control virus was also constructed for comparative analysis.

### Cell culture and treatment

MIN6 cells were obtained from the Cancer Research Institute of Central South University (Changsha, China) and maintained in Dulbecco’s modified Eagle’s medium (DMEM; Sigma, Oakville, Canada) supplemented with 16.7 mM glucose, 10% fetal bovine serum (FBS; Thermo Fisher Scientific, Waltham, MA, USA), 100 IU/mL penicillin, 50 µg/mL streptomycin, and 2 mM glutamine (all from Sigma Aldrich, St. Louis, MI, USA) in a 37 °C humidified incubator with 5% CO_2_. Cells in the exponential growth phase were initially exposed to ox-LDL (40 μg/mL; Yiyuanbiotech, Guangzhou, China) or LDL (40 μg/mL, Yiyuanbiotech, Guangzhou, China) for 24 h to induce lipotoxic injury and determine the decrease level of SCGN. Then, ox-LDL was chosen as the optimal lipid composition for use in the following experiments. To achieve SCGN overexpression, MIN6 cells were transfected with the pcDNA3.1-Scgn vector (rSCGN) using Lipofectamine^TM^ 3000 transfection reagent (Invitrogen, Carlsbad, USA). Following transfection, a stable cell line overexpressing SCGN was generated and subsequently identified. Conversely, SCGN knockdown experiments were conducted using siRNAs specific to *SCGN*. The transfection procedures and detailed siRNA sequences were described in our previous study [24].

### Cell viability and cell death assays

Cell viability (%) was assessed using the 3-(4,5-dimethylthiazole-2-yl)-2,5-diphenyltetrazole ammonium bromide (MTT) assay. Briefly, MIN6 cells were seeded into a 96-well cell culture plate and subjected to treatment with specific processing factors for 24 h. Afterward, 20 μL of MTT solution (5 mg/mL) was added to each well, and the cells were further incubated for 4 h. Following the incubation, the supernatant was discarded, and 150 μL of dimethyl sulfoxide was added to dissolve the crystal sediments in each well. The optical density was measured at 570 nm using a microplate reader (ELx800; BioTek, USA).

Pyroptotic cell death was assessed using Propidium Iodide (PI)/Hoechst 33342 double staining and lactate dehydrogenase (LDH) release assay. For PI/Hoechst 33342 double staining, MIN6 cells were seeded in 24-well plates overnight and treated with the corresponding processing factors for 24 h. After treatment, the cells were incubated with a mixture of Hoechst 33342 and PI for 20 min and then observed under a fluorescence microscope (Axioscope 5; Zeiss, Oberkochen, Germany) for imaging. For LDH release analysis, the cell culture supernatants were collected, and the LDH activity was determined using the LDH assay kit obtained from Nanjing Jiancheng Biology Engineering Institute (Nanjing, Jiangsu, China). To perform the LDH assay, a total of 25 μL of cell supernatant was mixed with 25 μL of substrate and incubated at 37 °C for 15 min. Subsequently, 25 μL of 2,4-dinitrophenylhydrazine was added to the samples, followed by another 15-minute incubation at 37 °C. Finally, 250 μL of a 0.4 mol/L NaOH solution was added, and the mixture was incubated at room temperature for 5 min. The absorbance of the samples was measured at 450 nm using a microplate reader.

### Animals and treatment

A total of twenty-four male C57BL/6 mice, aged between 12 to 16 weeks, were randomly divided into four groups (*n* = 6 for each group): ND group (normal chow diet), HFD group (high-fat diet), HFD + AAV-9 group (Mice fed an HFD and injected with the AAV-9 empty vector) and HFD + AAV-9-SCGN group (Mice fed an HFD and injected with the AAV-9 containing the *SCGN* gene). During the experimental period, the mice were assigned to either an HFD or an ND for a duration of 16 weeks. The diet treatment was initiated at the beginning of the experiment. Two weeks after the diet treatment commenced, AAV-9 infection was administered to the mice via tail vein injection at a dose of 2.2 × 10^11^ vg (viral genomes) per mouse. At the end of the 16-week treatment period, the mice were humanely sacrificed, and samples of their serum and pancreas were collected for further analysis and investigation. Our procedures were conducted in compliance with the guidelines of the National Health and Medical Research Council of China and approved by the animal ethics review board of the First Affiliated Hospital, University of South China.

### Assessment of biological parameters

The serum levels of fasting blood glucose (FBG), cholesterol (TC), triglyceride (TG), and low-density lipoprotein (LDL) in mice were measured using an automatic biochemical instrument (Cobas 8000, Roche diagnostics, Mannheim, Germany), following the manufacturer’s instructions.

### ELISA assay

After treating the mice, serum samples were collected from each group. The concentrations of SCGN and the fasting insulin (FINS) levels in the serum were determined using ELISA kits purchased from Cloud-clone Corp, Ltd. (Wuhan, China) and Beyotime Biotechnology (Shanghai, China), respectively. The manufacturer’s instructions were followed to perform the assays.

### Glucose tolerance test and insulin resistance index calculation

FBG and FINS levels were tested as described above. Using the obtained FBG and FINS results, the homeostatic model assessment of insulin resistance (HOMA-IR) index was calculated. For the glucose tolerance test (GTT), mice were fasted for 6 h and then intraperitoneally injected with 1 g/kg of glucose. Blood glucose levels were measured at 0, 30, 60, and 120 min after the injection.

### Islet isolation

The mice were euthanized by CO_2_ anesthetization, and the pancreas was then carefully exposed through anatomical dissection. The digestion process was initiated by injecting collagenase V solution through the bile duct. The pancreas was minced or mechanically disrupted and incubated with collagenase in a 37 °C water bath for 15 min. After the incubation, the digest reaction was terminated by adding a cold Hanks’ balanced salt solution (HBSS) with 1% FBS. The dissociated tissue was then filtered through a mesh to separate the intact islets from other cell debris and smaller tissue fragments. The filtered suspension containing the islets was layered onto a density gradient solution and centrifuged. This separation technique allowed the islets to migrate to a specific layer in the gradient based on their density. Careful pipetting was used to collect the islets from the density gradient layer. The isolated islets were cultured in a DMEM medium, supplemented with nutrients and growth factors.

### Western blot analysis

MIN6 cells or the islets were lysed using RIPA lysis buffer (Beyotime Biotechnology, Shanghai, China). The protein concentration in the lysates was determined using a BCA protein assay kit (Beyotime, Shanghai, China). Total proteins were separated by SDS-PAGE and transferred onto polyvinylidene fluoride (PVDF) membranes. Subsequently, the PVDF membranes were blocked in 5% fat-free milk in 0.1% Tris-buffered saline (TBS)-Tween (TBST) for 2 h at room temperature. The membranes were then incubated separately with primary antibodies against SCGN (1:2000), TXNIP (1:1000), NLRP3 (1:1000), IL-1β (1:1000), Caspase-1 (1:1000), GSDMD (1:1000) or GAPDH (1:5000). After washing, the membranes were incubated with the appropriate secondary antibody (1:5000) for 1 h at room temperature. Finally, the protein bands were visualized using an efficient chemiluminescence kit (Beyotime, Shanghai, China), captured using a Fusion Solo S optical system (Vilber Lourmat, France), and further analyzed using ImageJ software.

### Real-time quantitative PCR

RNA was extracted from harvested cells and islets using the TRIzol reagent (Thermo Fisher Scientific, Inc.). The isolated RNA was then reverse transcribed using MMLV Reverse Transcription Reagents (Promega Corporation) at 37 °C for 1 h. Quantitative real-time PCR (qRT-PCR) was performed using the PRISM 7500HT Real-Time PCR System (Applied Biosystems, USA) with SYBR Green Master Mix and gene-specific primers (Supplementary Table 1). Gapdh was used as an endogenous control, and the relative gene expression data were analyzed using the 2^−ΔΔCt^ method.

### Immunofluorescence staining

MIN6 cells were seeded in six-well plates, with a cover slide placed in each well. After overnight incubation to allow cell attachment, the cells were treated with ox-LDL, SCGN siRNA, ox-LDL in combination with rSCGN, or the corresponding control factors. The intracellular protein distribution and the levels of SCGN, Caspase-1, and ChREBP were assessed using immunocytofluorescence staining, following the protocols outlined in our previous study [35]. The primary antibodies against SCGN, Caspase-1, and ChREBP were diluted at a ratio of 1:200, and the fluorescein isothiocyanate-conjugated secondary antibody was diluted at a ratio of 1:300 (cat. no. 711‑545‑1500; Jackson ImmunoResearch Laboratories, Inc.). The fluorescence signal was visualized using a fluorescence microscope (Axioscope 5; Zeiss, Oberkochen, Germany).

For pancreatic tissues, paraffin-embedded mouse pancreas sections were prepared. The sections were de-paraffinized and then blocked in PBS containing 0.5% Triton and 5% normal goat serum for 2 h. Prior to incubation with primary antibodies, antigen retrieval was carried out by using a citrate buffer. The following day, the samples were incubated with the appropriate fluorogenic secondary antibodies and DAPI for 1 h. Images were captured using a fluorescence microscope. The exposure settings remained unchanged throughout the image acquisition process. Staining was analyzed using ImageJ software.

### In silico protein interaction prediction

The protein crystal structures of SCGN and ChREBP were obtained from the Protein Database (PDB) with a resolution of less than 3 Å. In order to analyze the binding capability of these proteins, a docking analysis was performed using the HDOCK server (https://hdock.phys.hust.edu.cn). The resulting protein interactions were further analyzed and visualized using the PyMol software. The possibility of protein interaction was mainly evaluated based on the docking score and confidence score. Specifically, a docking score below -200 or a confidence score above 0.7 indicates a high likelihood of binding between the two molecules [36].

### Co-immunoprecipitation assay

MIN6 cells were lysed using RIPA lysis buffer (Beyotime Biotechnology, Shanghai, China) to extract proteins. The protein concentration in the lysates was determined using a BCA protein assay kit (Beyotime, Shanghai, China). Next, the ChREBP antibody or SCGN antibody was added to the lysate and incubated overnight at 4 °C. The lysate was then incubated with protein A/G beads for 3–5 h. The sediment collected from the beads was washed three times with lysis buffer and subsequently boiled for 5 min. The boiled samples were centrifuged again, and the resulting supernatant was used for subsequent western blot analysis.

### Statistical analysis

All data were presented as mean ± standard deviation (SD) and were derived from a minimum of three independent biological replicates. Statistical analysis was conducted using GraphPad Prism 8 (GraphPad Software Inc., San Diego, CA). The student’s *t*-test was used for pairwise comparisons, while one-way analysis of variance (ANOVA) was employed for multiple comparisons. A *p* value of less than 0.05 was considered statistically significant.

### Supplementary information

Supplementary Table 1

Supplementary figures

Original full length western blots

Original full length western blots

Original full length western blots

## Data Availability

The dataset generated during the current study is available from the corresponding author upon reasonable request.
