# Supplementary material for: The downregulation of SCGN induced by lipotoxicity promotes NLRP3-mediated β-cell pyroptosis
Source: Cell Death Discov. 2024 Jul 27;10:340. doi: 10.1038/s41420-024-02107-y (PMC11283536; doi:10.1038/s41420-024-02107-y)
Supplement: Supplementary file 1 — Supplementary Table 1 [file 41420_2024_2107_MOESM1_ESM.docx]

**Supplementary Tab. 1 specific** **PCR primer sequence for gene amplification**

| **Gene** | **Forward primer** | **Reverse primer** |
| --- | --- | --- |
| *Scgn* | 5′-CCCAGAAGTGGATGGATTTG-3′ | 5′-GTTGGGGATCAGGGGTTTAT-3′ |
| *Caspase-1* | 5′-ACAAGGCACGGGACCTATG-3′ | 5′-TCCCAGTCAGTCCTGGAAATG-3′ |
| *Txnip* | 5′- AGCCTGCAGGAAATGAAGCA-3′ | 5′- ACCTGCCTACTGATTGCCAC-3′ |
| *Nlrps* | 5′-GACCGTGAGGAAAGGACCAG-3′ | 5′-GGCCAAAGAGGAATCGGACA-3′ |
| *GSDMD* | 5′-ATGCCATCGGCCTTTGAGAAA-3′ | 5′-AGGCTGTCCACCGGAATGA-3′ |
| *IL-1β* | 5′-GCAACTGTTCCTGAACTCAACT-3′ | 5′-ATCTTTTGGGGTCCGTCAACT-3′ |
