# Supplementary material for: The downregulation of SCGN induced by lipotoxicity promotes NLRP3-mediated β-cell pyroptosis
Source: Cell Death Discov. 2024 Jul 27;10:340. doi: 10.1038/s41420-024-02107-y (PMC11283536; doi:10.1038/s41420-024-02107-y)
Supplement: Supplementary file 2 — Supplementary figures [file 41420_2024_2107_MOESM2_ESM.zip › Supplementary figure file/Supplementary figure legends.docx]

**Fig. S1** **Lipotoxicity downregulates SCGN expression in MIN6 cells.**

**A-B** Western blot analysis was performed to determine the protein expression levels of SCGN. GAPDH was used as a loading control. n = 3 per group; **P*<0.05.

**Fig. S2 Screening and identification of the efficiency of three SCGN siRNAs.**

**A** Real-time quantitative PCR was performed to measure the mRNA expression levels of SCGN in the transfected cells. **B,C** Western blot analysis was performed to determine the protein expression levels of SCGN. GAPDH was used as a loading control. n = 3 per group; ***P*<0.01 compared to the control group.
