# Supplementary figures and images for: The downregulation of SCGN induced by lipotoxicity promotes NLRP3-mediated β-cell pyroptosis
Source: Cell Death Discov. 2024 Jul 27;10:340. doi: 10.1038/s41420-024-02107-y (PMC11283536; doi:10.1038/s41420-024-02107-y)

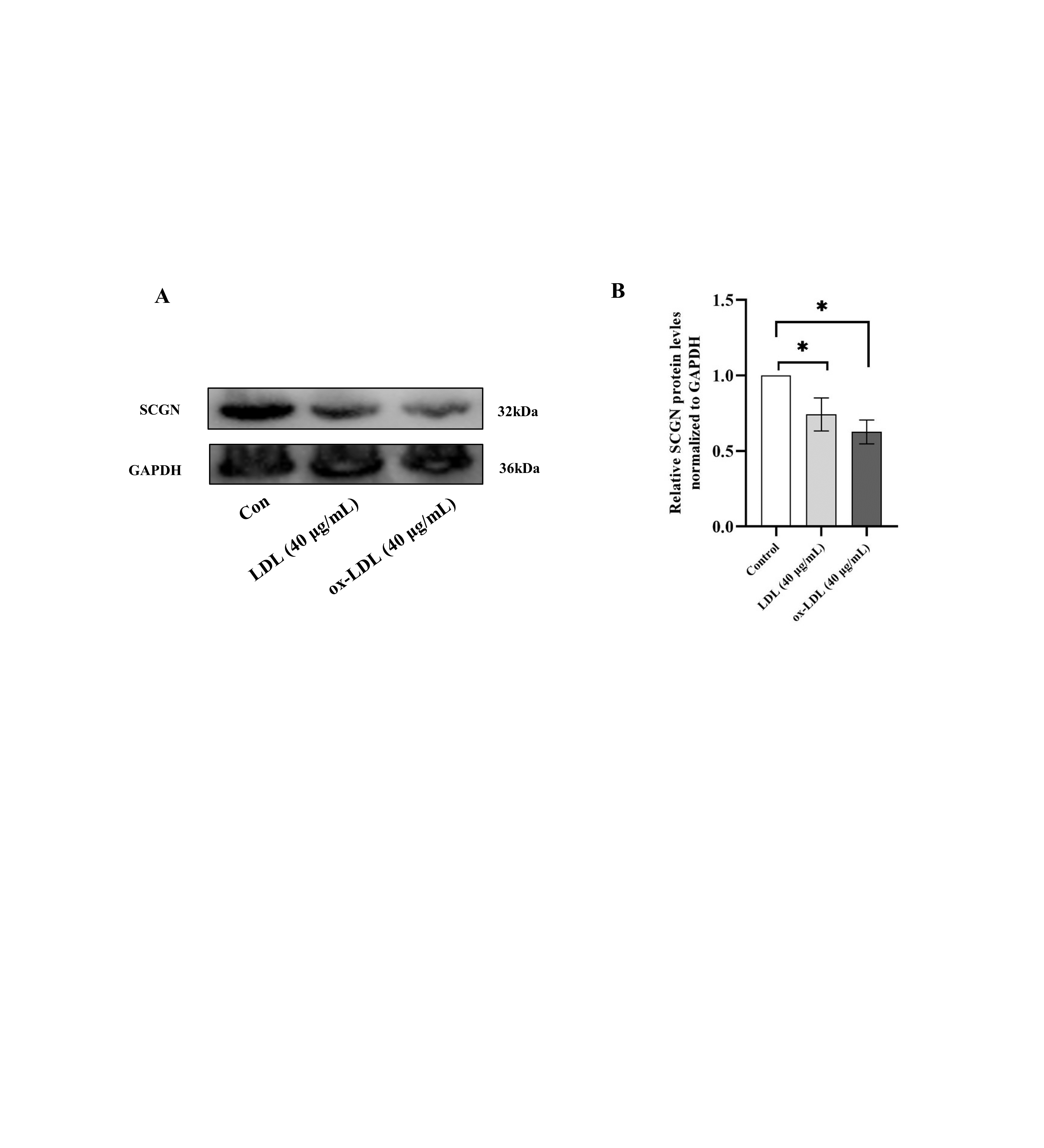

Supplement: Supplementary file 2 — Supplementary figures [file 41420_2024_2107_MOESM2_ESM.zip › Supplementary figure file/Figure S1.PNG]

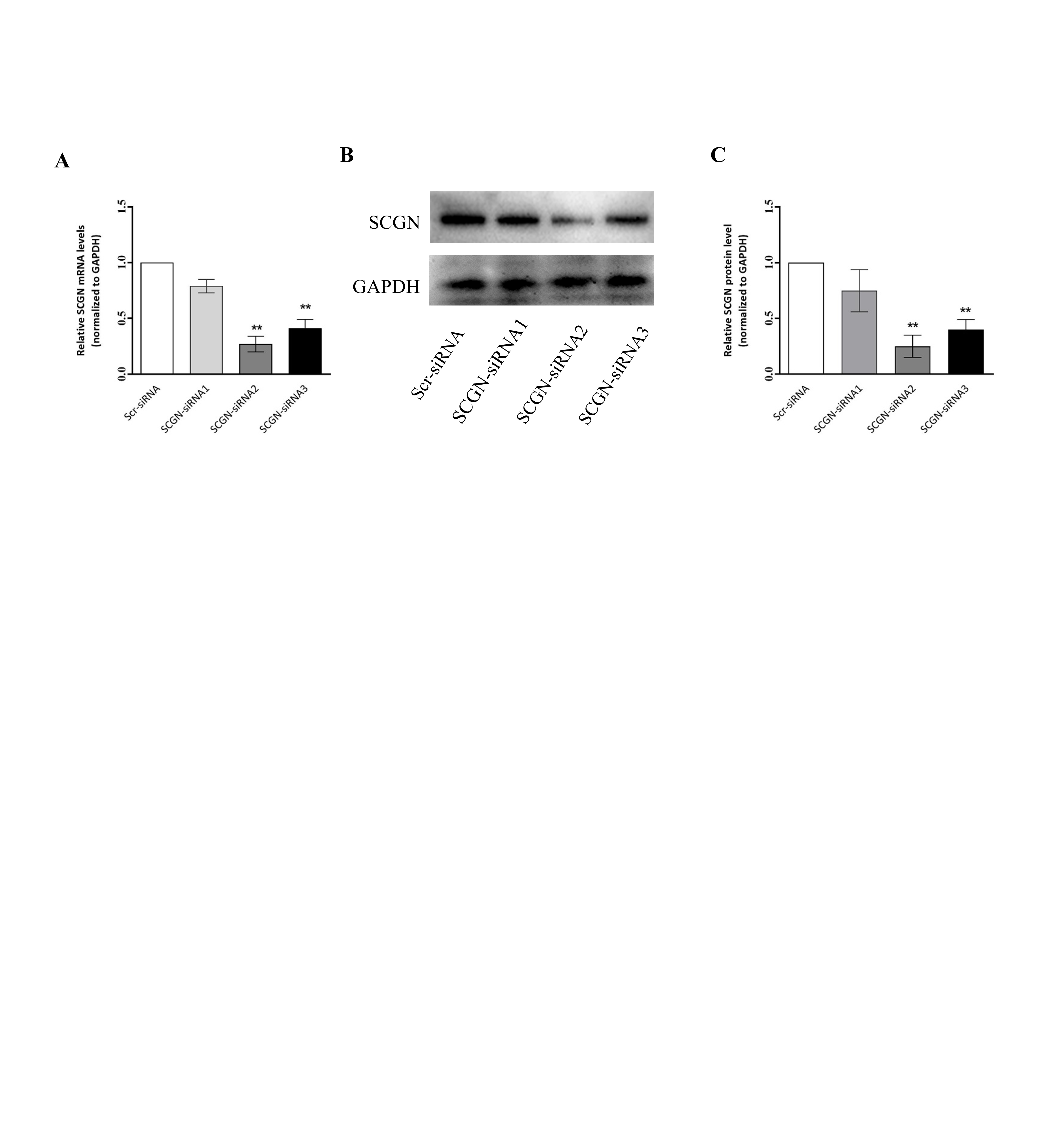

Supplement: Supplementary file 2 — Supplementary figures [file 41420_2024_2107_MOESM2_ESM.zip › Supplementary figure file/Figure S2.PNG]
