# Supplementary material for: The downregulation of SCGN induced by lipotoxicity promotes NLRP3-mediated β-cell pyroptosis
Source: Cell Death Discov. 2024 Jul 27;10:340. doi: 10.1038/s41420-024-02107-y (PMC11283536; doi:10.1038/s41420-024-02107-y)

SCGN-1 (CON LDL ox-LDL palmitic acid)

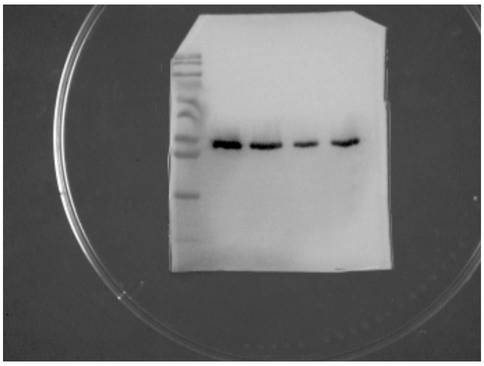

GAPDH-1 (CON LDL ox-LDL palmitic acid)

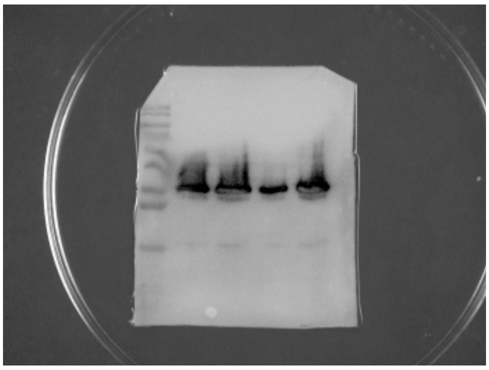

(Paper selection chart)

SCGN-2 (CON LDL ox-LDL palmitic acid)

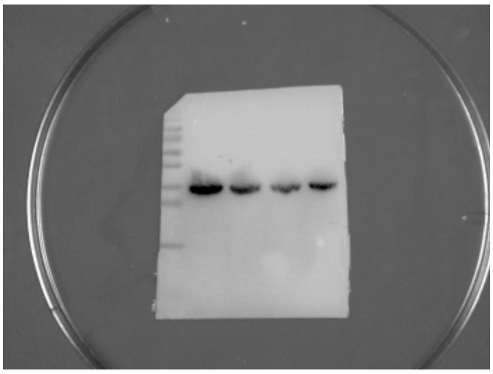

GAPDH-2 (CON LDL ox-LDL palmitic acid)

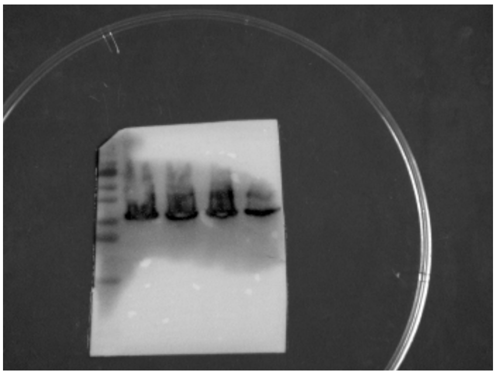

SCGN-3 (CON LDL ox-LDL palmitic acid)

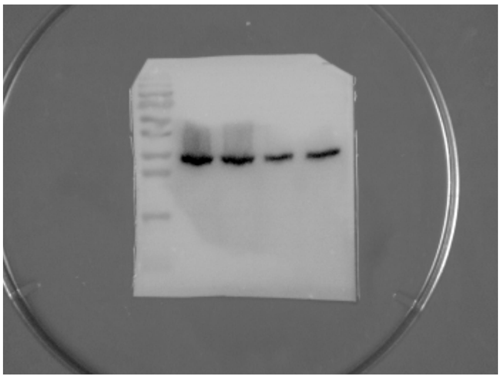

GAPDH-3 (CON LDL ox-LDL palmitic acid)

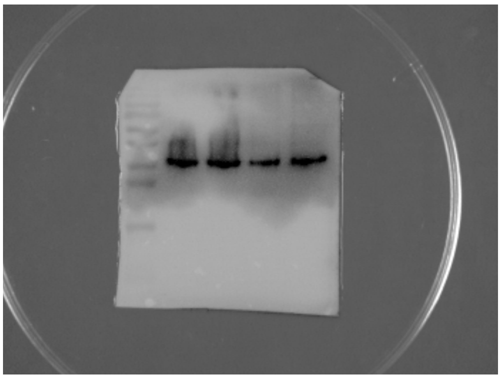

Supplement: Supplementary file 3 — Original full length western blots [file 41420_2024_2107_MOESM3_ESM.pdf]
