# Supplementary material for: The downregulation of SCGN induced by lipotoxicity promotes NLRP3-mediated β-cell pyroptosis
Source: Cell Death Discov. 2024 Jul 27;10:340. doi: 10.1038/s41420-024-02107-y (PMC11283536; doi:10.1038/s41420-024-02107-y)
Supplement: Supplementary file 4 — Original full length western blots [file 41420_2024_2107_MOESM4_ESM.pdf]

ChREBP precipitation SCGN

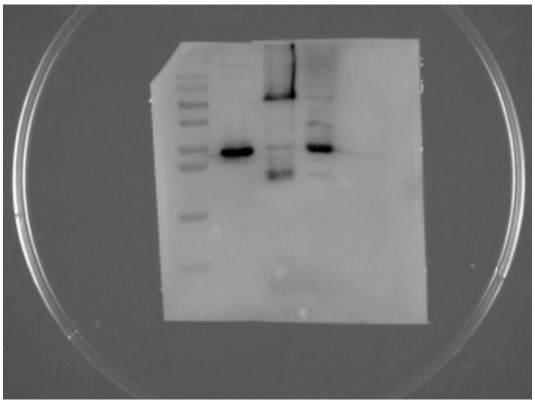

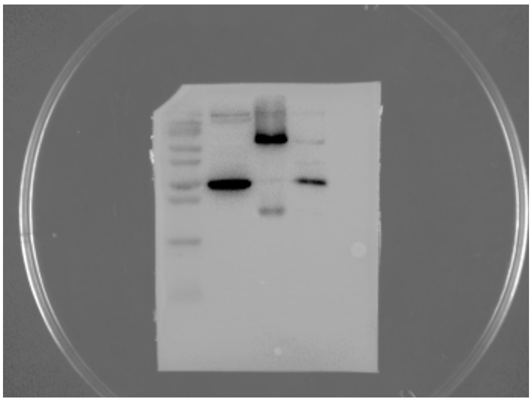

ChREBP precipitation SCGN (control)

ChREBP precipitation SCGN (ox-LDL)

SCGN precipitation ChREBP

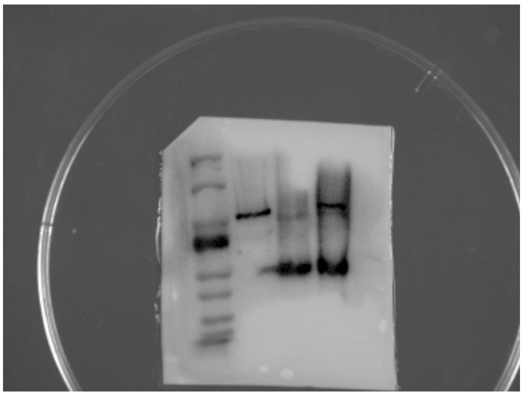

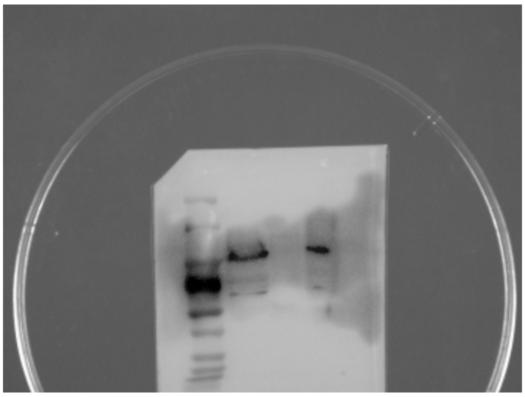

SCGN precipitation ChREBP (control)

SCGN precipitation ChREBP (ox-LDL)
