# Supplementary material for: The downregulation of SCGN induced by lipotoxicity promotes NLRP3-mediated β-cell pyroptosis
Source: Cell Death Discov. 2024 Jul 27;10:340. doi: 10.1038/s41420-024-02107-y (PMC11283536; doi:10.1038/s41420-024-02107-y)

Figure 1 (ox-LDL 0, 10, 20, 40)
First time

SCGN

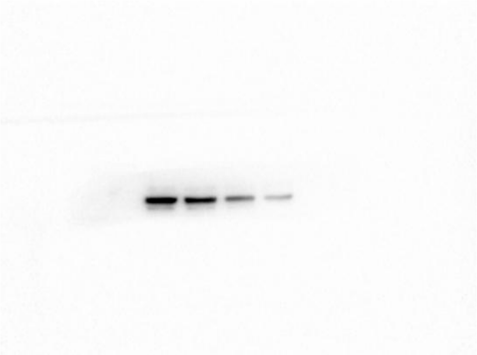

TXNIP

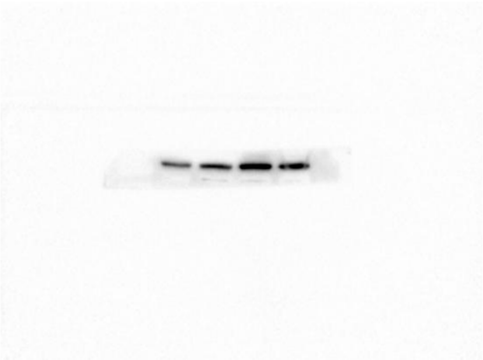

NLRP3

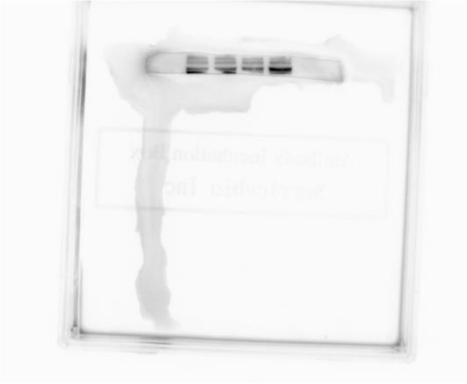

Pro-Caspase-1

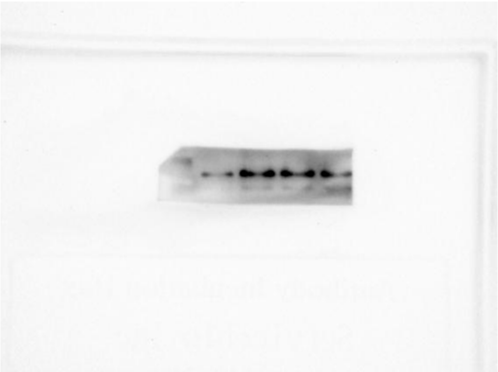

Caspase-1

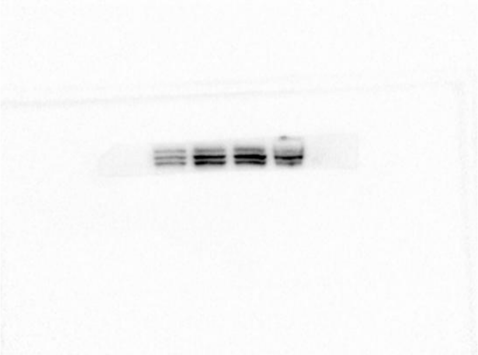

GSDMD-NT

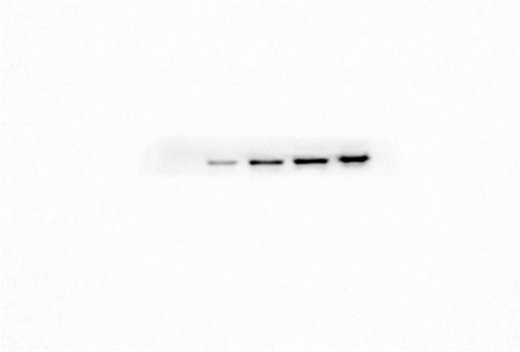

IL-1β

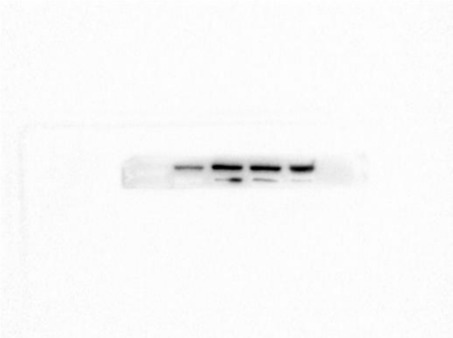

GAPDH

Replication 1

SCGN

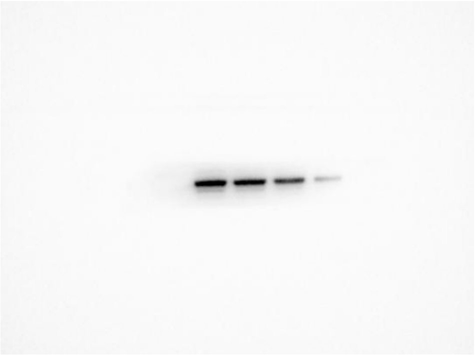

TXNIP

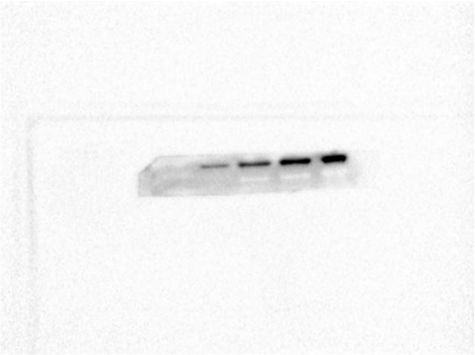

NLRP3

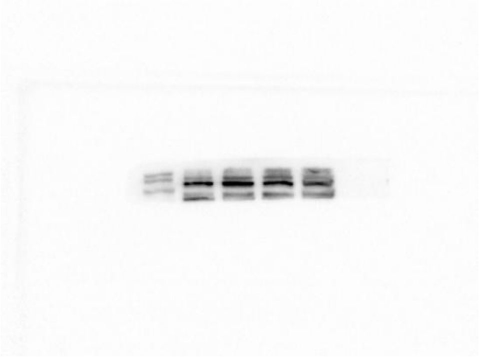

Pro-Caspase-1

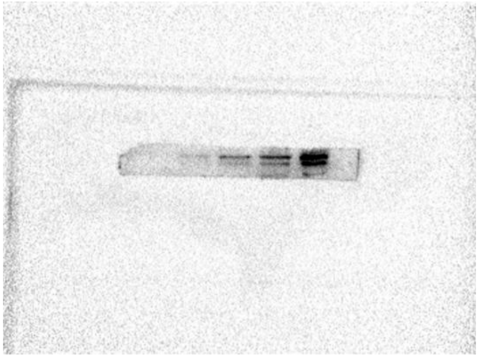

Caspase-1

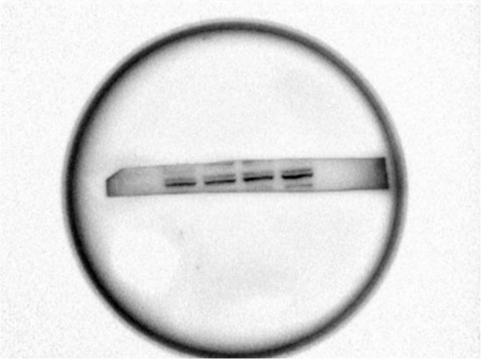

GSDMD-NT

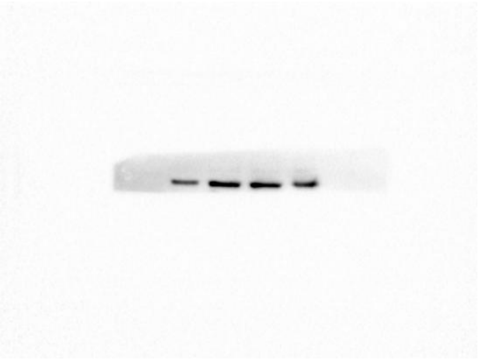

IL-1β

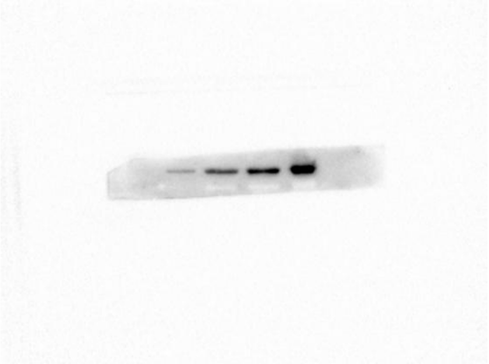

GAPDH

Replication 2

SCGN

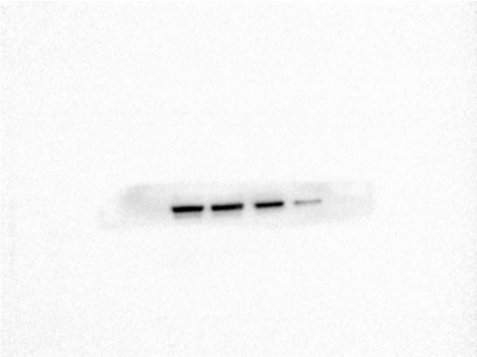

TXNIP

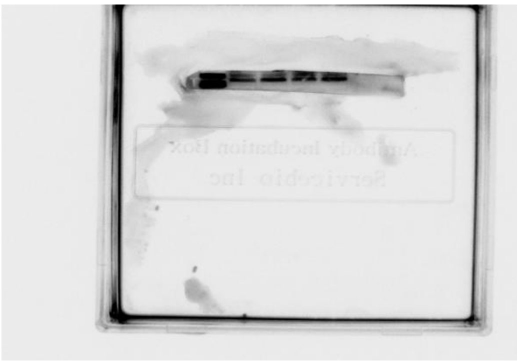

NLRP3

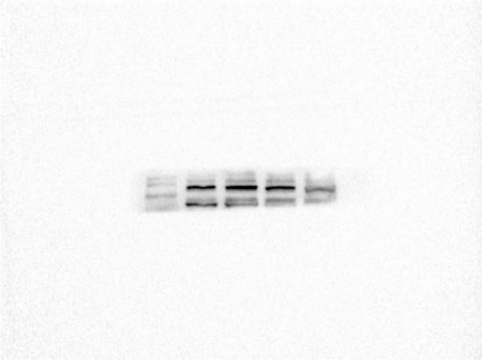

Pro-Caspase-1

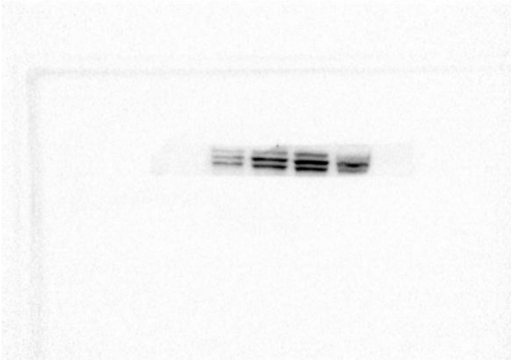

Caspase-1

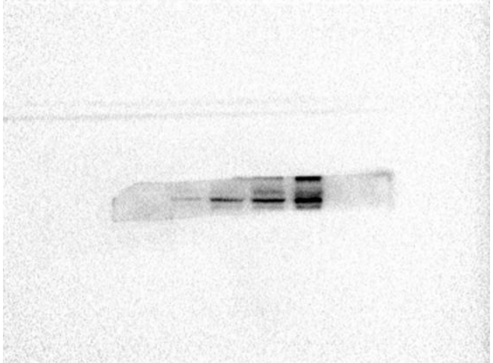

GSDMD-NT

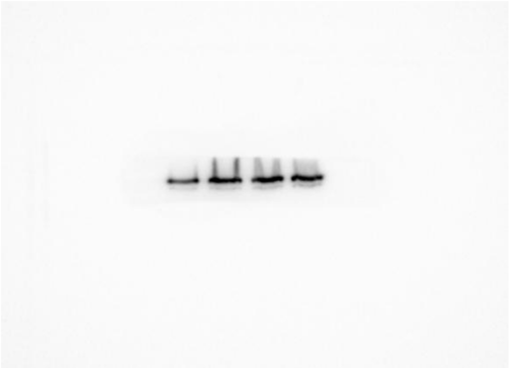

IL-1β

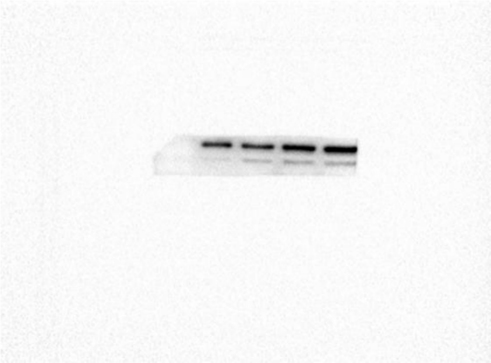

GAPDH

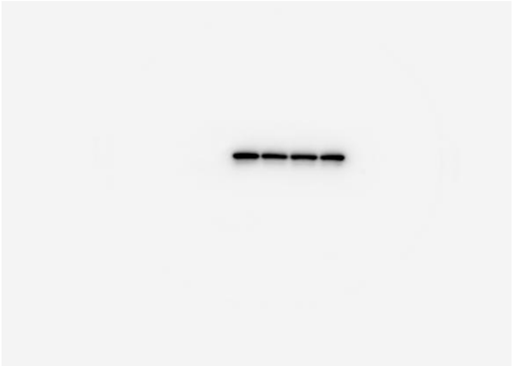

Figure 2 (HFD)

First time

SCGN

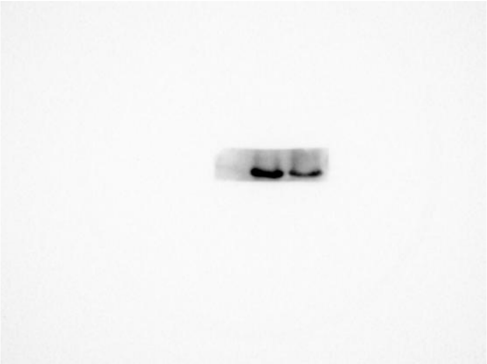

Caspase-1

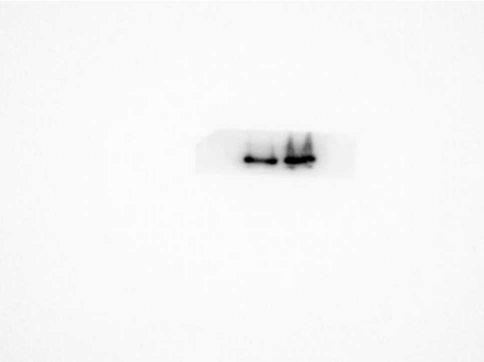

GAPDH

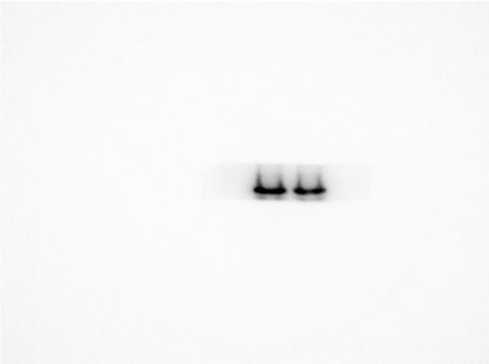

Replication 1

SCGN

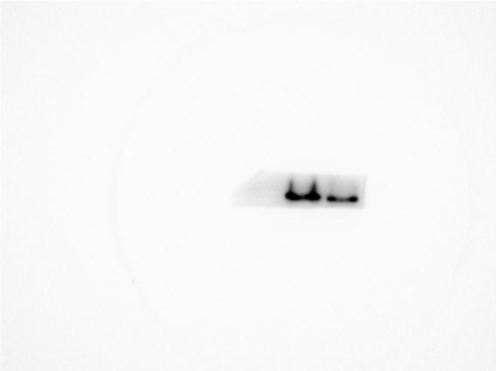

Caspase-1

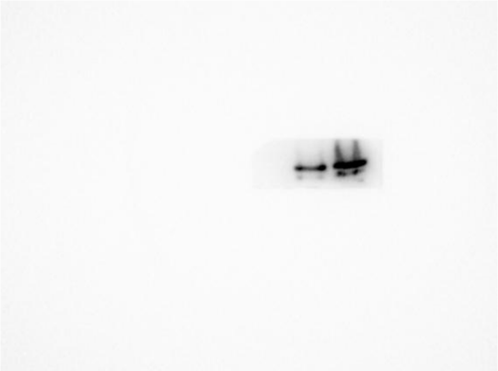

GAPDH

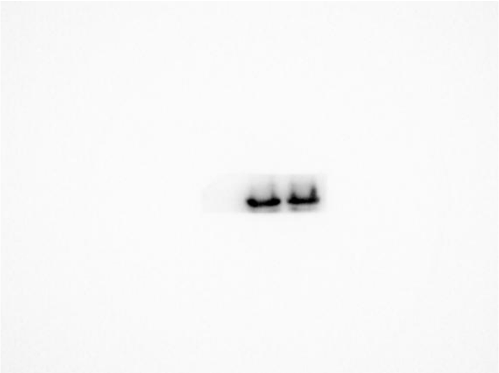

Replication 2

SCGN

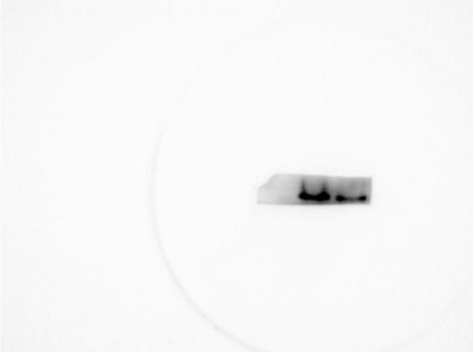

Caspase-1

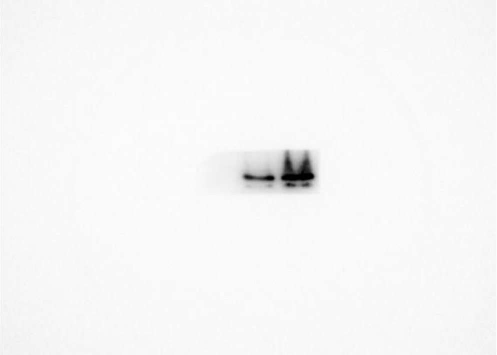

GAPDH

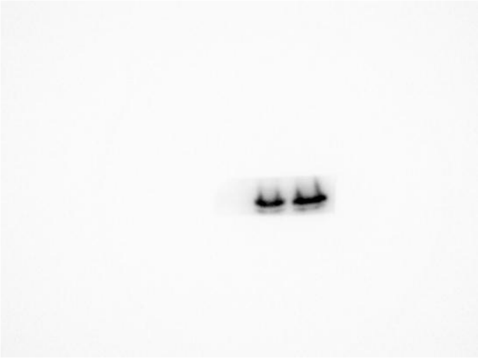

Figure 3 (con, scr-siRNA, siRNA, ox-LDL)

First time

SCGN

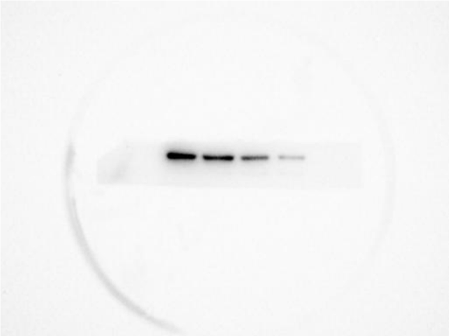

TXNIP

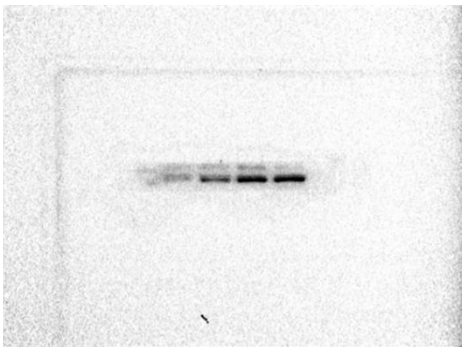

NLRP3

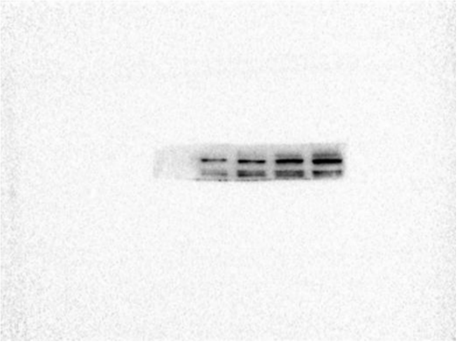

Pro-Caspase-1

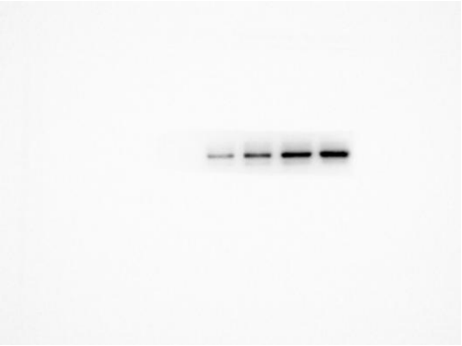

Caspase-1

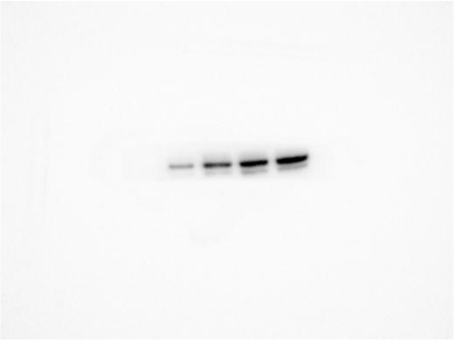

GSDMD-NT

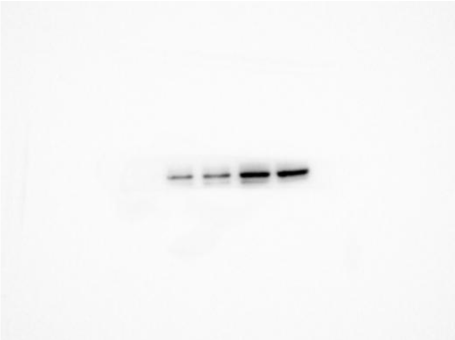

IL-1β

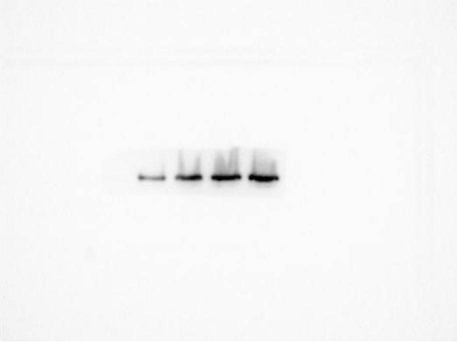

GAPDH

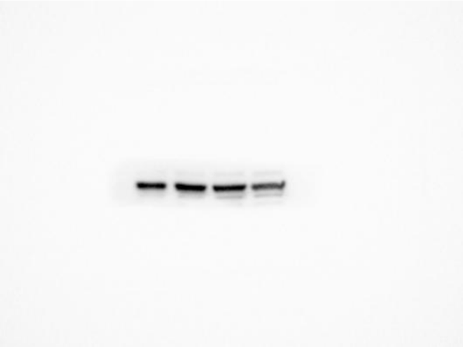

Replication 1

SCGN

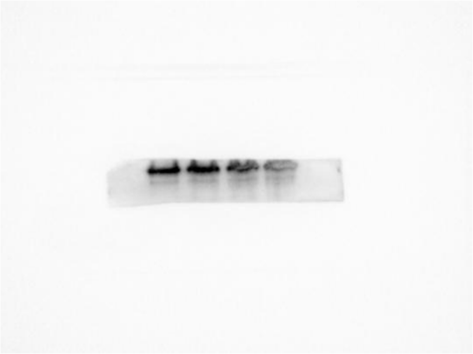

TXNIP

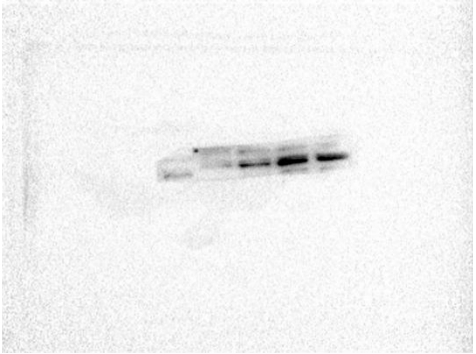

NLRP3

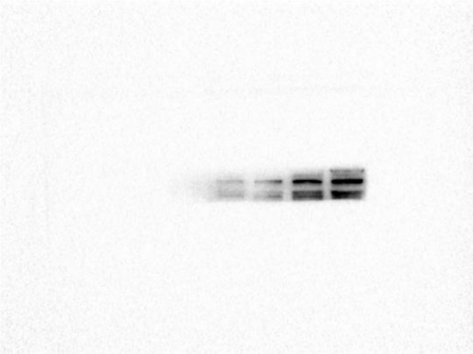

Pro-Caspase-1

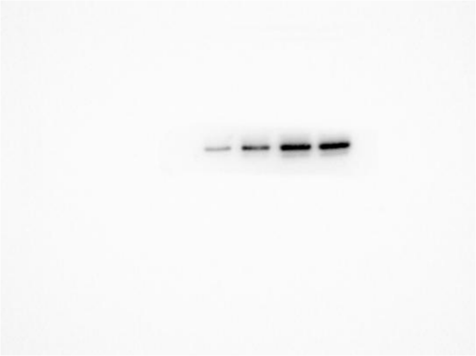

Caspase-1

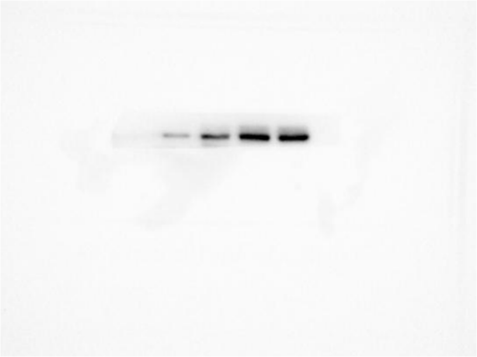

GSDMD-NT

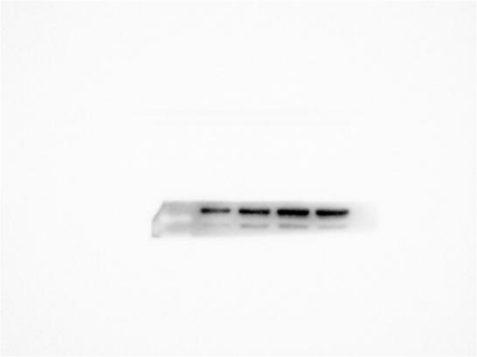

IL-1β

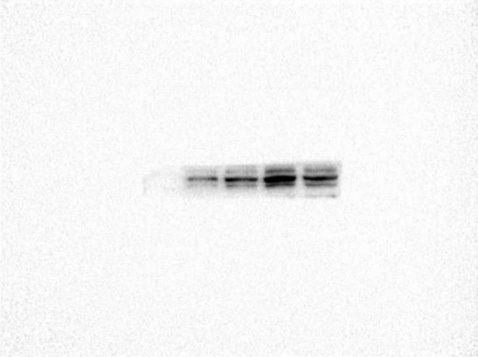

GAPDH

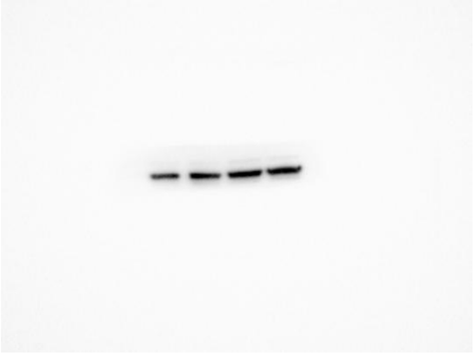

Replication 2

SCGN

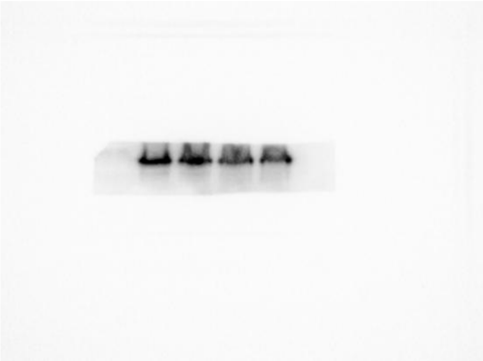

TXNIP

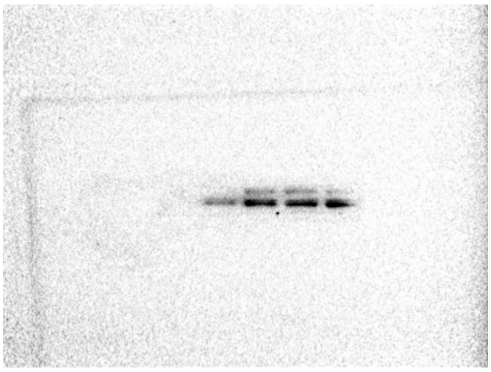

NLRP3

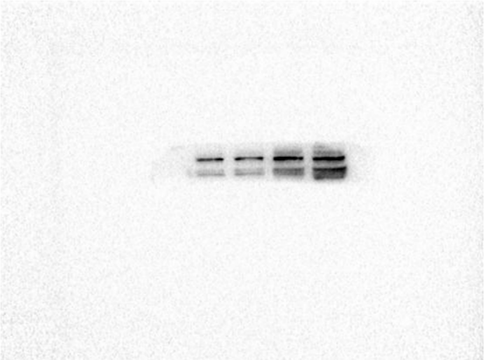

Pro-Caspase-1

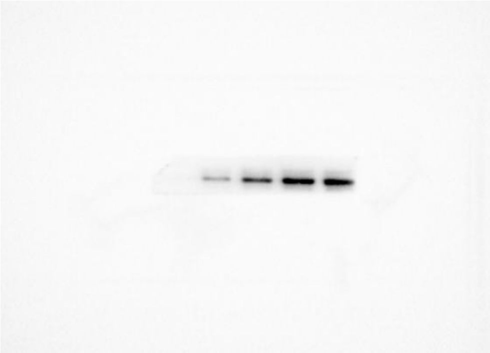

Caspase-1

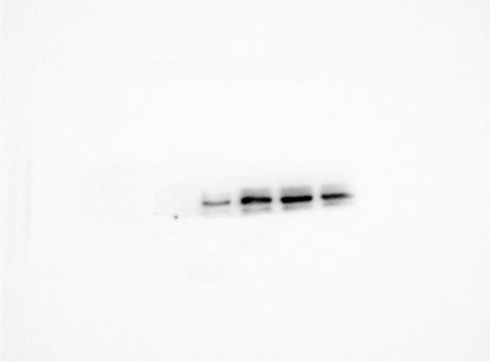

GSDMD-NT

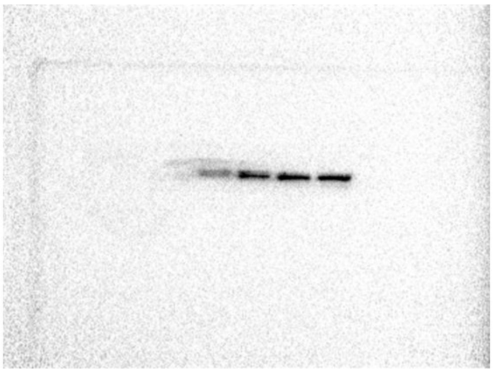

IL-1β

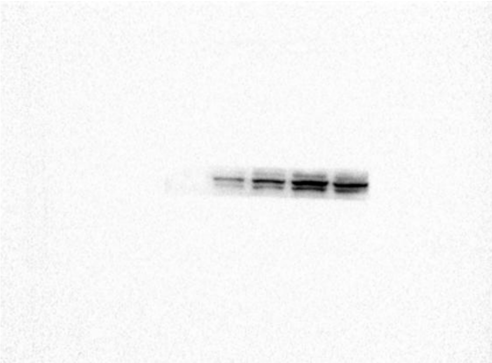

GAPDH

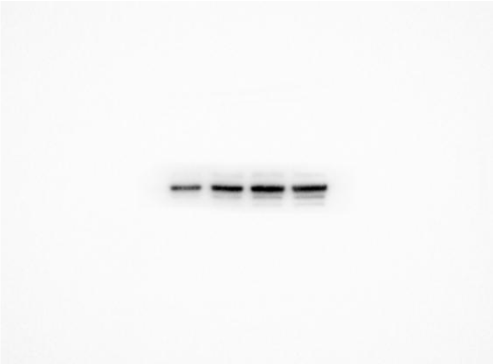

Figure　4 (con, ox-LDL, ox-LDL + Vector, ox-LDL + SCGN)

First time

SCGN

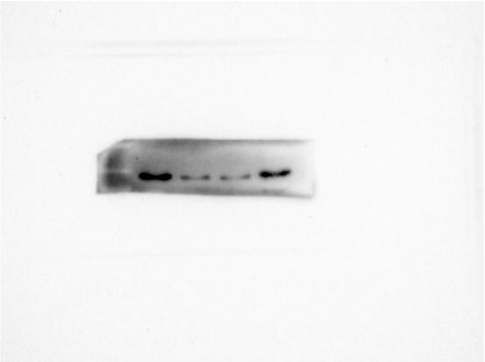

TXNIP

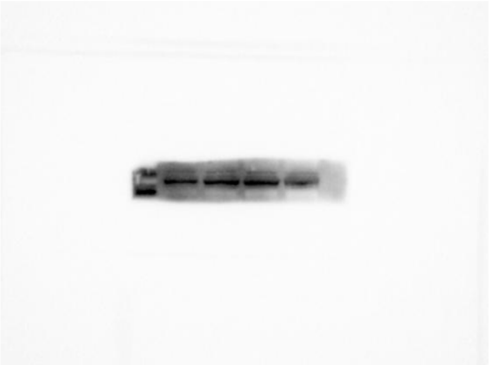

NLRP3

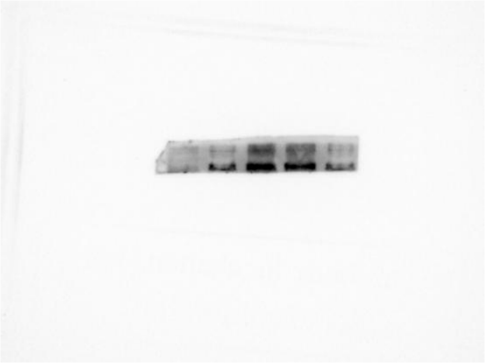

Pro-Caspase-1

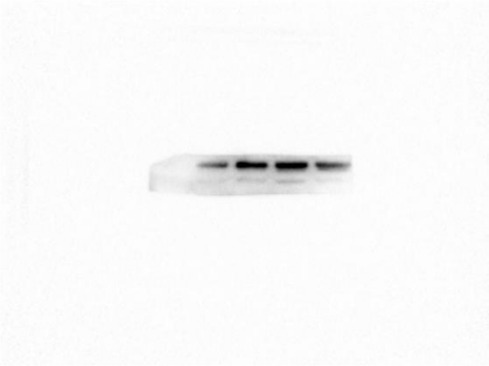

Caspase-1

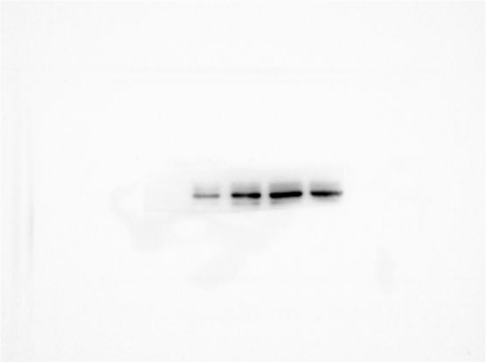

GSDMD-NT

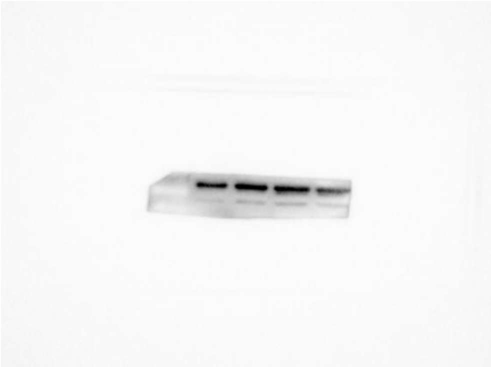

IL-1β

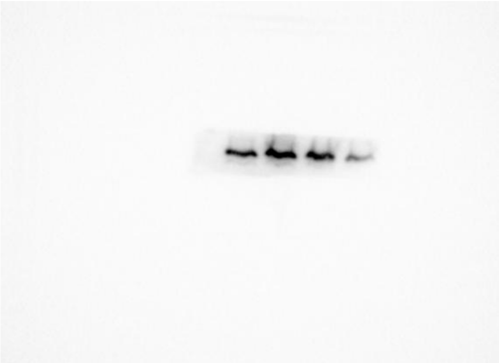

GAPDH

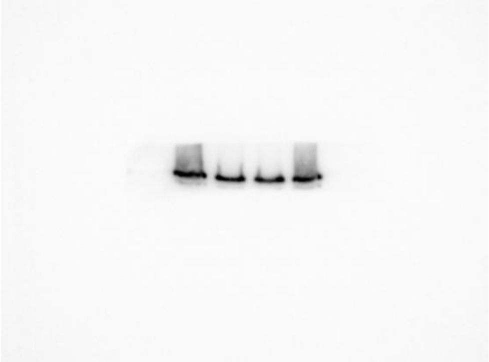

Replication 1

SCGN

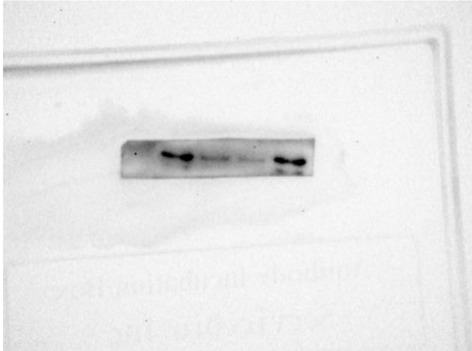

TXNIP

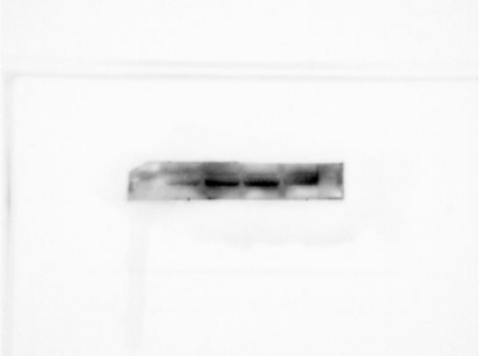

NLRP3

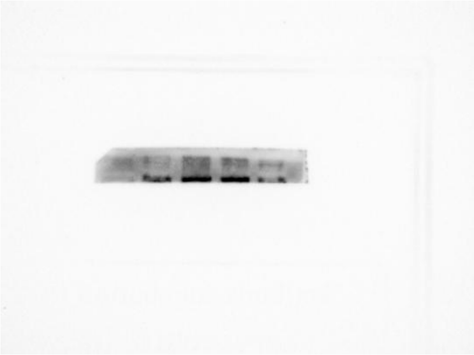

Pro-Caspase-1

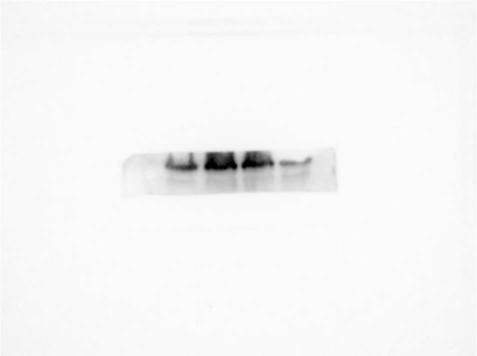

Caspase-1

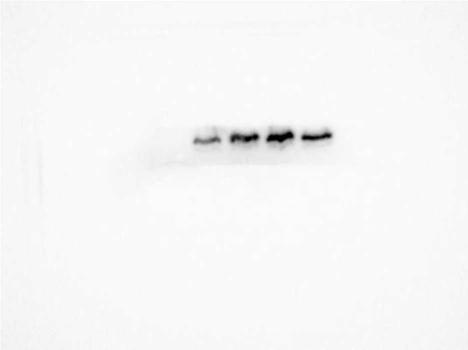

GSDMD-NT

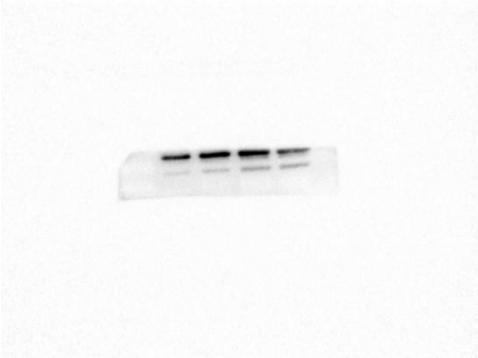

IL-1β

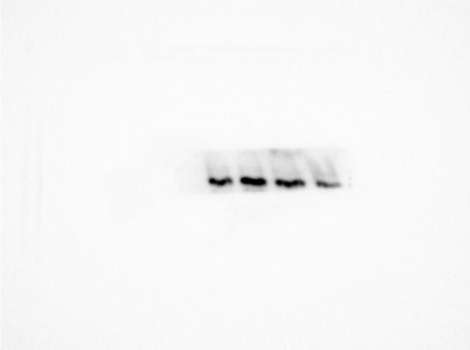

GAPDH

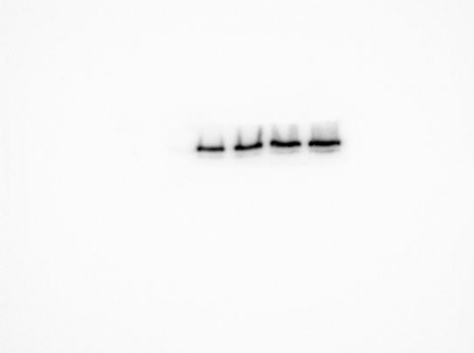

Replication 2

SCGN

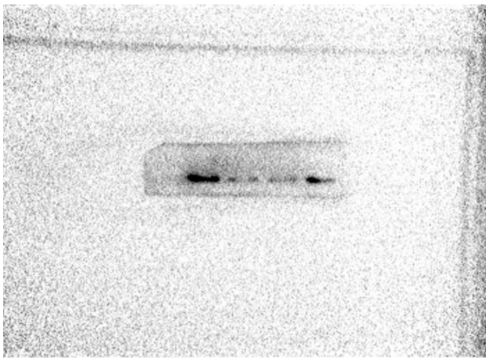

TXNIP

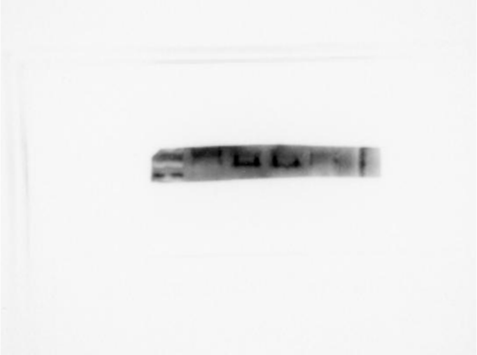

NLRP3

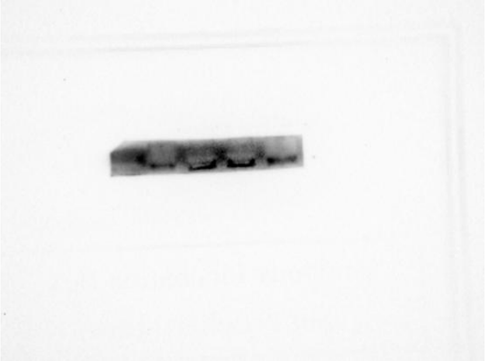

Pro-Caspase-1

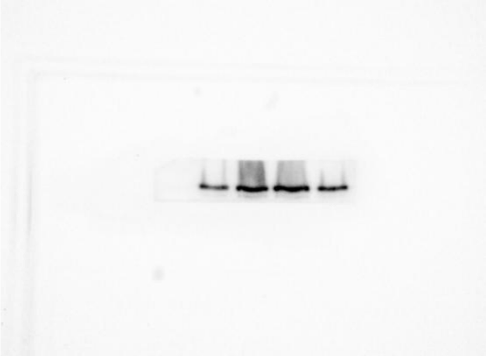

Caspase-1

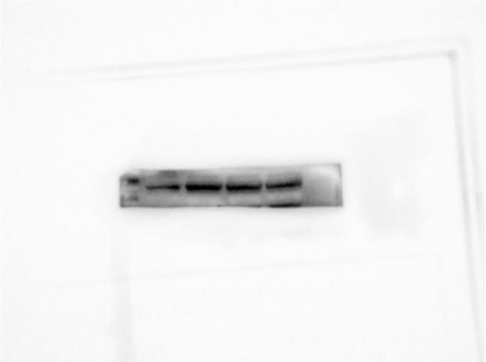

GSDMD-NT

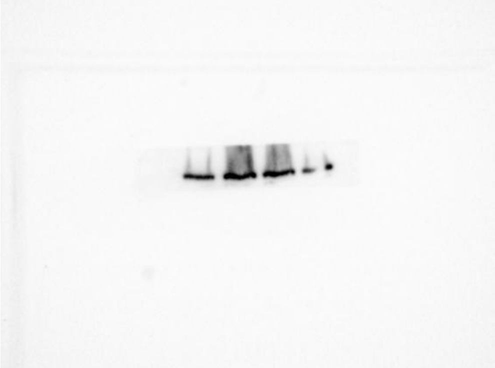

IL-1β

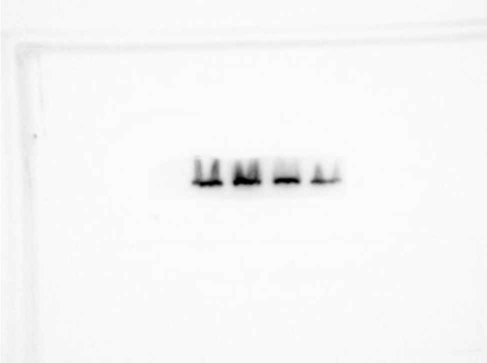

GAPDH

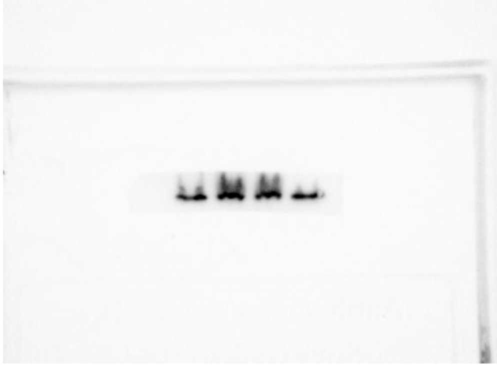

Figure 5 (HFD + AAV9-SCGN)

First time

SCGN

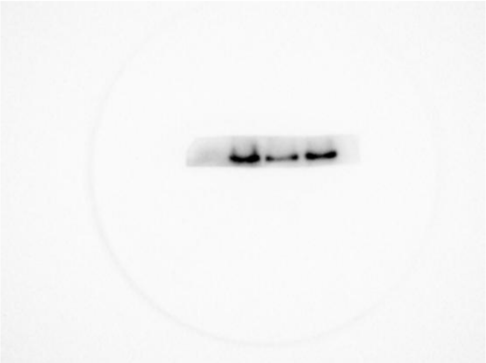

Caspase-1

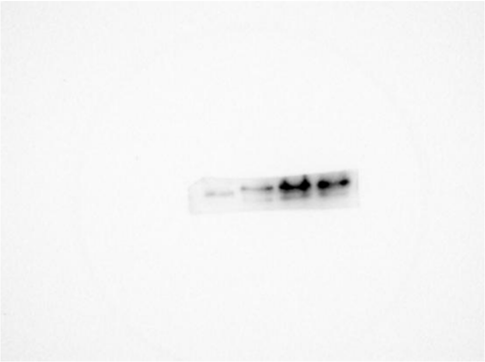

GAPDH

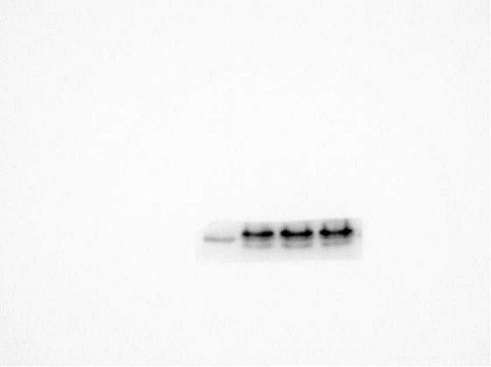

Replication 1

SCGN

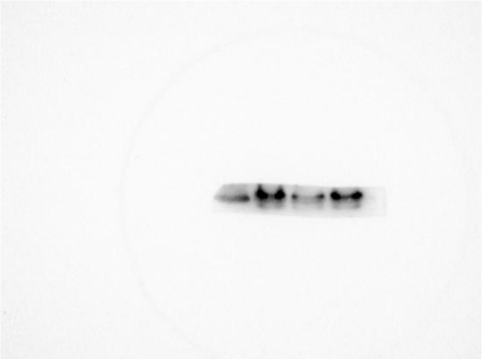

Caspase-1

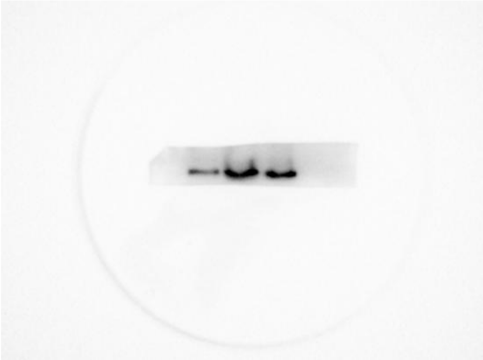

GAPDH

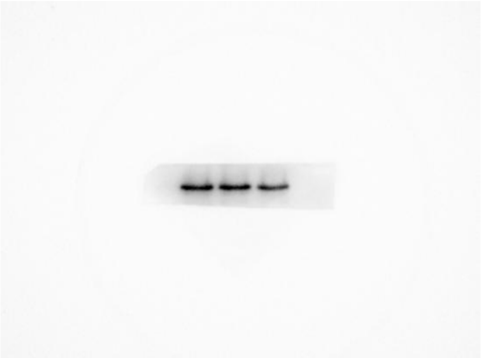

Replication 2

SCGN

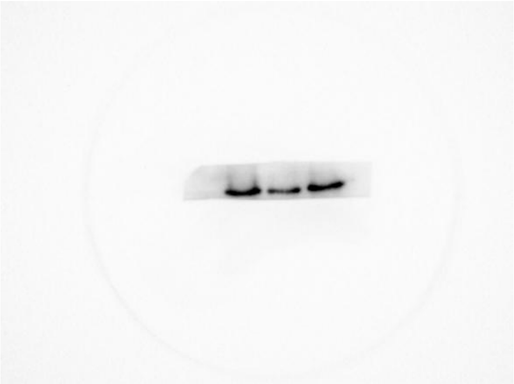

Caspase-1

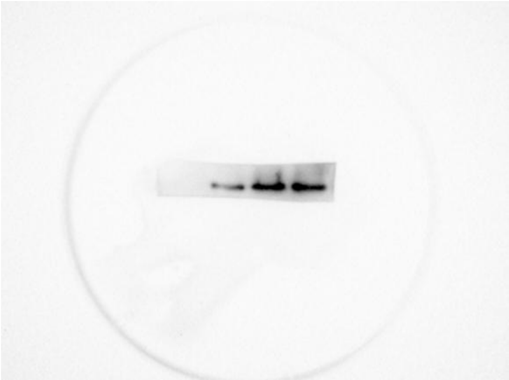

GAPDH

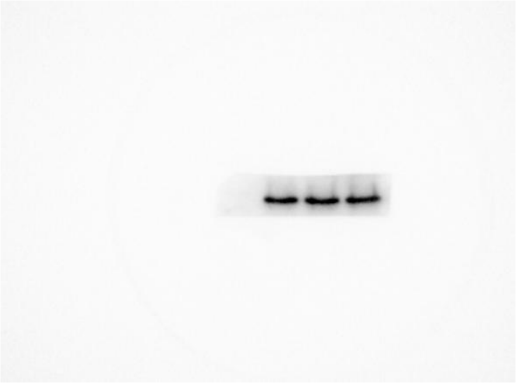

Figure 6 (con, scrRNA, siRNA, ox-LDL)

First time (Cytoplasm)

ChREBP

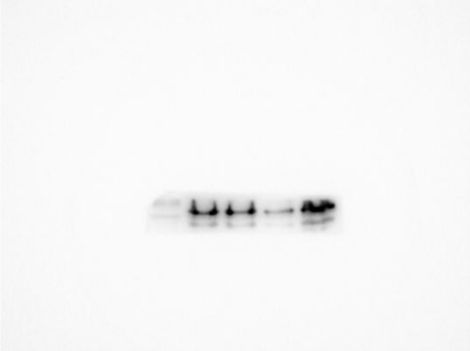

GAPDH

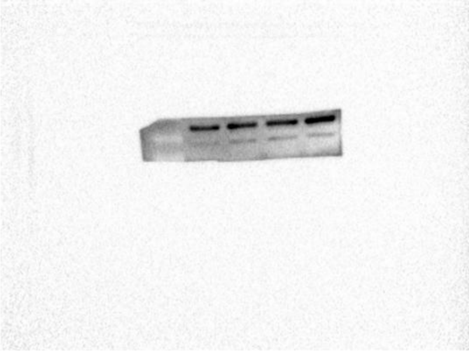

GAPDH

Replication 1

ChREBP

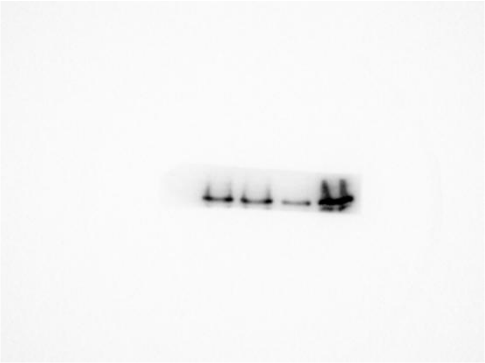

GAPDH

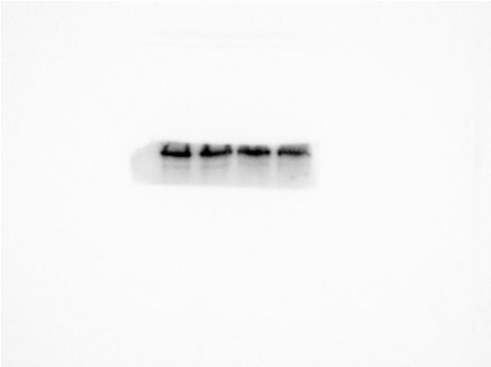

GAPDH

Replication 2

ChREBP

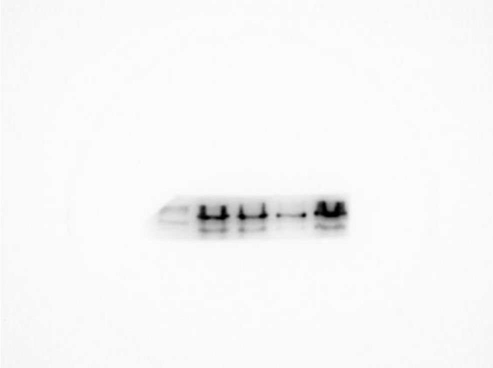

GAPDH

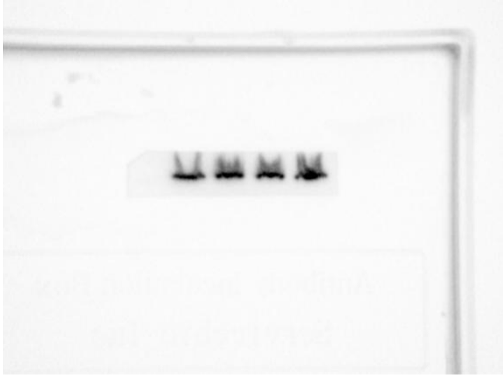

First time (Nucleus)

ChREBP

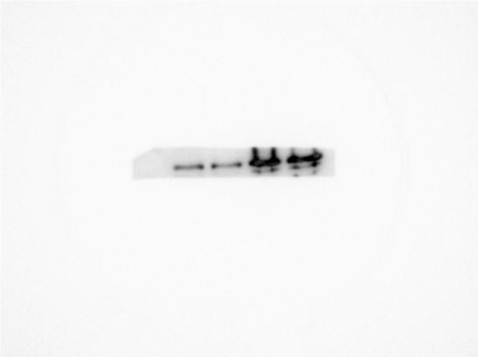

Histon H1

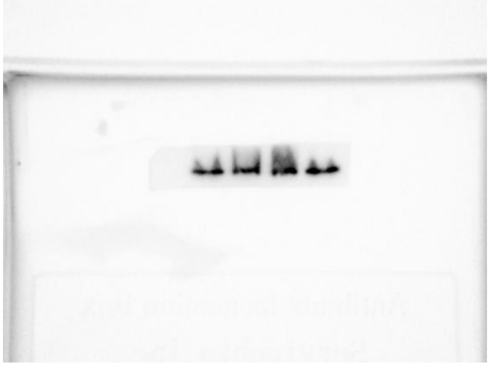

GAPDH

Replication 1

ChREBP

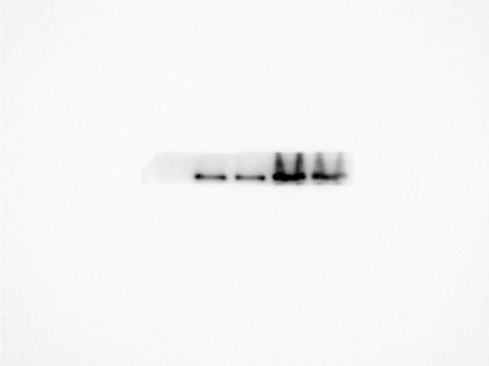

Histon H1

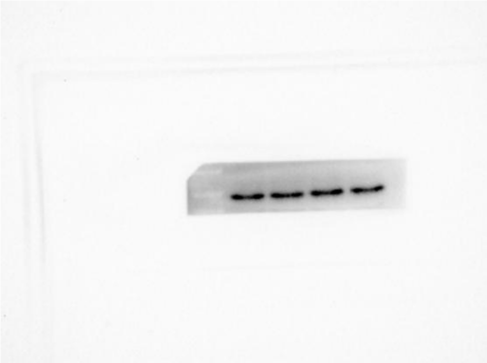

GAPDH

Replication 2

ChREBP

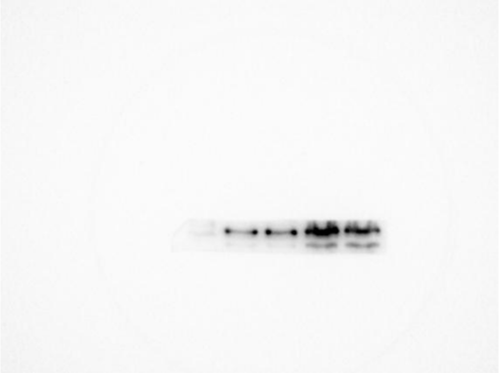

Histon H1

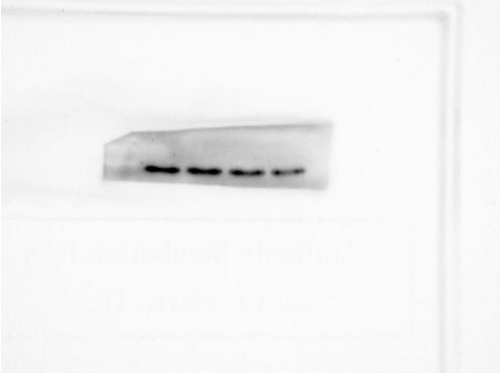

Figure 6 (con, LDL, LDL+Vector, LDL+SCGN)

First time (Cytoplasm)

ChREBP

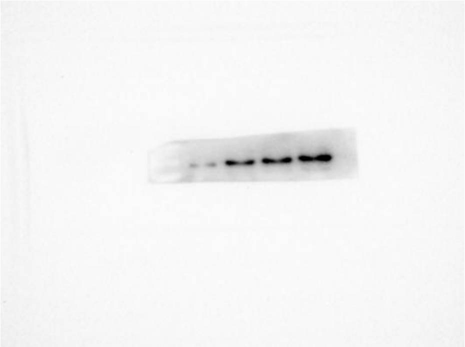

GAPDH

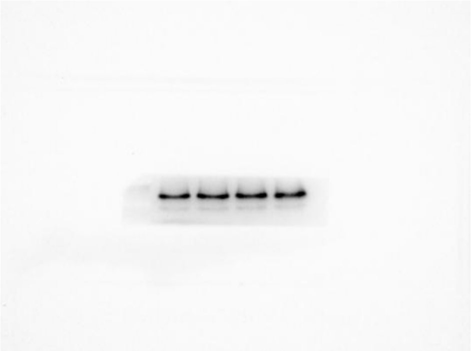

GAPDH

Replication 1

ChREBP

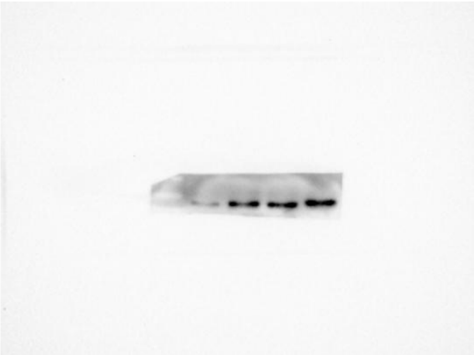

GAPDH

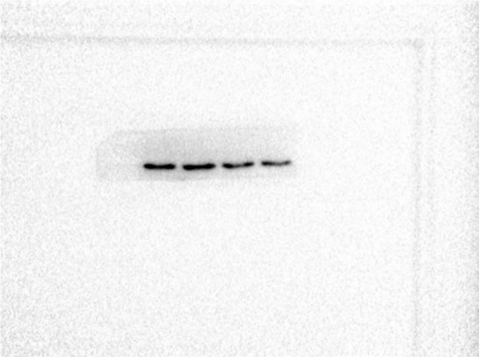

GAPDH

Replication 2

ChREBP

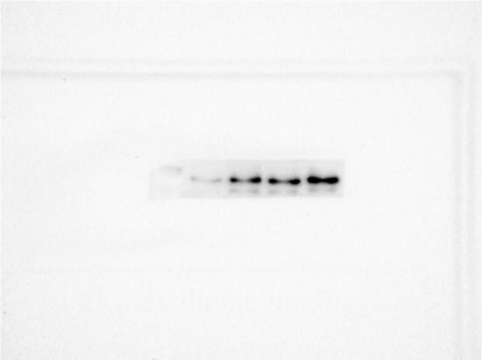

GAPDH

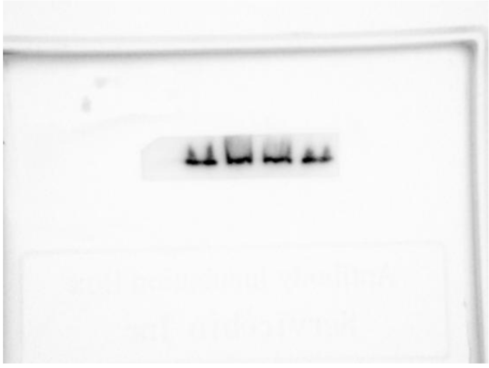

First time (Nucleus)

ChREBP

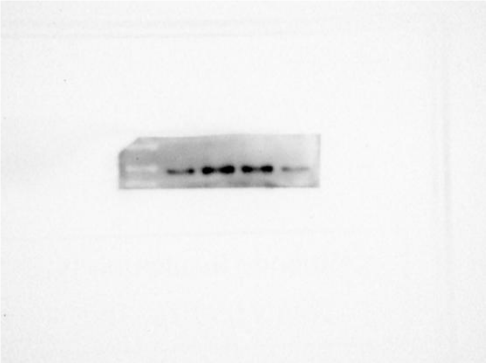

Histon H1

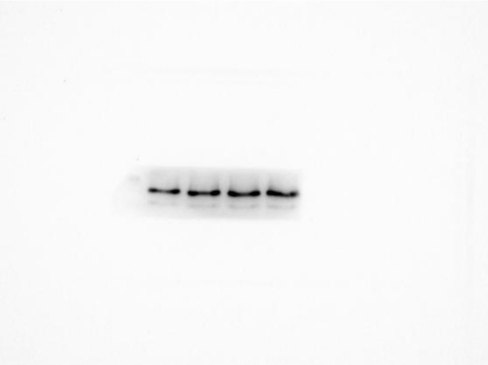

GAPDH

Replication 1

ChREBP

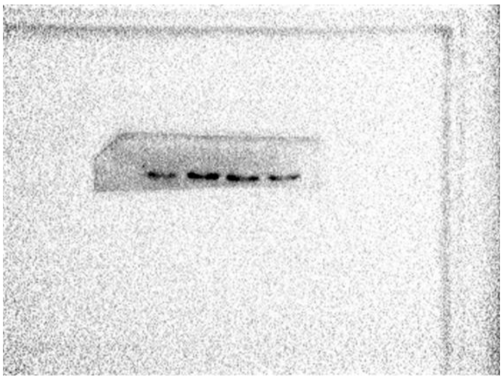

Histon H1

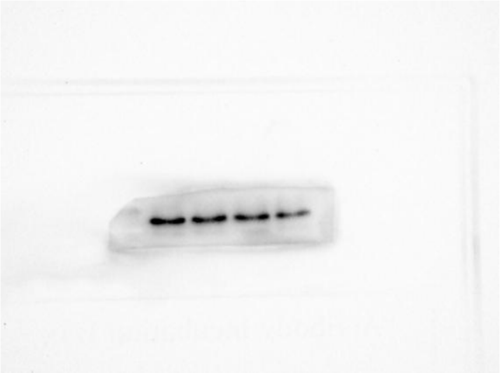

GAPDH

Replication 2

ChREBP

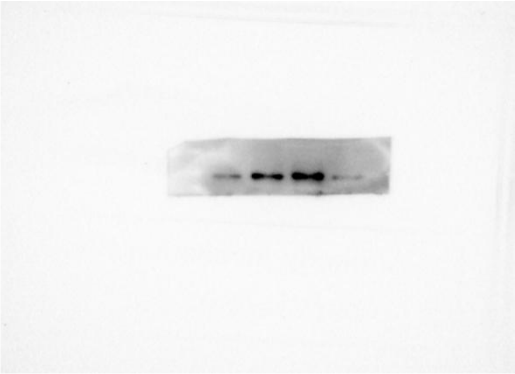

Histon H1

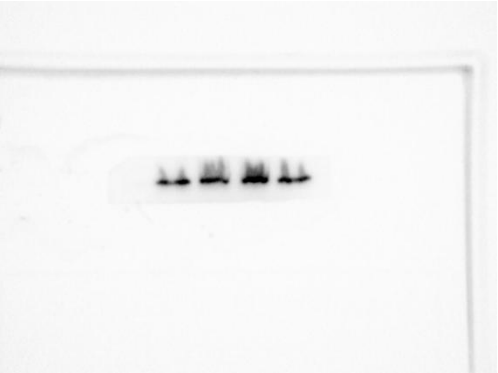

Supplement: Supplementary file 5 — Original full length western blots [file 41420_2024_2107_MOESM5_ESM.pdf]
